# Impact of Anionic Dopants on H Atom Uptake at Polyoxovanadate-Alkoxide Surfaces

**DOI:** 10.1021/acs.inorgchem.5c02940

**Published:** 2025-08-27

**Authors:** M. Rebecca A. Walls, Rachel L. Meyer, William W. Brennessel, Ellen M. Matson

**Affiliations:** Department of Chemistry, 6927 University of Rochester, Rochester, New York 14627, United States

## Abstract

Proton-coupled electron transfer (PCET) is an important mechanism that defines the reactivity of H atom equivalents at reducible metal oxide (MO_*x*_) surfaces. To better understand structure–function properties that dictate the thermochemistry and kinetics of PCET at MO_*x*_ surfaces, our group has employed polyoxovanadate-alkoxide (POV-alkoxide) complexes as molecular models of extended materials. In this work, we investigate the influence of anionic dopants on PCET reactivity in POV-alkoxides. We present the synthesis and characterization of two anion-substituted POV-ethoxides, [V_6_O_6_X­(OC_2_H_5_)_12_]^−^ (X = Cl or SCN). Reactivity of these assemblies with a potent H atom transfer reagent, 9,10-dihydrophenazine, in acetonitrile (MeCN) affords formation of the 2e^–^/2H^+^ reduced species, [V_6_O_6_X­(MeCN)­(OC_2_H_5_)_12_]^−^. The identity of the (pseudo)­halide dopant influences the rate of the reaction, wherein the thiocyanate-substituted species exhibits H atom uptake at rates 2× faster than its chloride congener, and 5× faster than the fully oxygenated assembly, [V_6_O_7_(OC_2_H_5_)_12_]^−^. Collectively, these results provide insight into the role the identify of the dopant plays in controlling the kinetics of H atom uptake/transfer at the surfaces of MO_*x*_.

## Graphical abstract

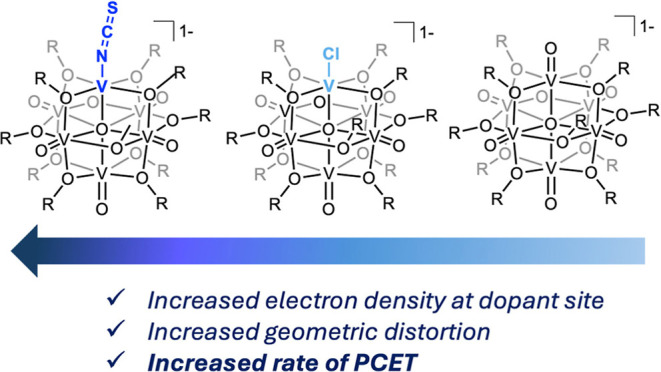

## Introduction

Proton-coupled electron transfer (PCET) is a key process in catalysis, energy storage, and energy conversion reactions at the surface of redox-active transition metal oxides (MO_*x*_).^1–5^ PCET can occur through a stepwise proton or electron led mechanism, generating charged intermediates, or through a concerted proton electron transfer (CPET) event.^3,5,6^ These pathways are related through the Bronsted acidity (p*K*_a_), redox potential, and bond dissociation free energy (BDFE) of the surface bound H atom.^6,7^ Modulations to these properties has been achieved through structural modifications of MO_*x*_, for example, doping a metal oxide lattice with cationic or anionic defects.^8–13^

In many examples, enhanced PCET reactivity has been noted at the surface of MO_*x*_ in the presence of anions, such as chloride or fluoride.^14–19^ In these systems, post reaction analysis of the material generally reveals an incorporation of these anions into the lattice, consistent with in situ formation of halide dopants. This has since prompted direct investigations of materials prepared with low levels of Cl or F inclusion for comparison with pristine materials. The enhanced reactivity and catalytic performance of anion doped materials has been attributed to multiple factors, including lattice distortion and charge compensation in metal valency, which generate more active metal-oxide sites adjacent to the dopant.

To gain further understanding of PCET at MO_*x*_ surfaces, our group has employed polyoxovanadate-alkoxides (POV-alkoxides) as solution-phase, atomically precise models of extended redox-active structures.^20–23^ These Lindqvist-type assemblies mimic a portion of the MO_*x*_ lattice, with bridging alkoxide ligands to promote solubility in organic media and in situ characterization via ^1^H NMR spectroscopy. Modifications to the parent hexavanadate structure has been investigated, resulting in the elucidation of structure–function relationships that define thermodynamics and kinetics of H atom uptake at POV-alkoxide surfaces. For example, our group has described dramatic changes in the rate of PCET to POV-alkoxides featuring O atom defect sites and cationic dopants (Figure 1).^21,22,24–27^ Similar to MO_*x*_ materials, both O atom defects and cationic dopants (i.e., Ti­(IV), Nb­(V)) yield enhanced kinetics for PCET as compared to the pristine structure, attributed to relief of geometric strain and enhanced reducibility, respectively.

**Figure 1:**
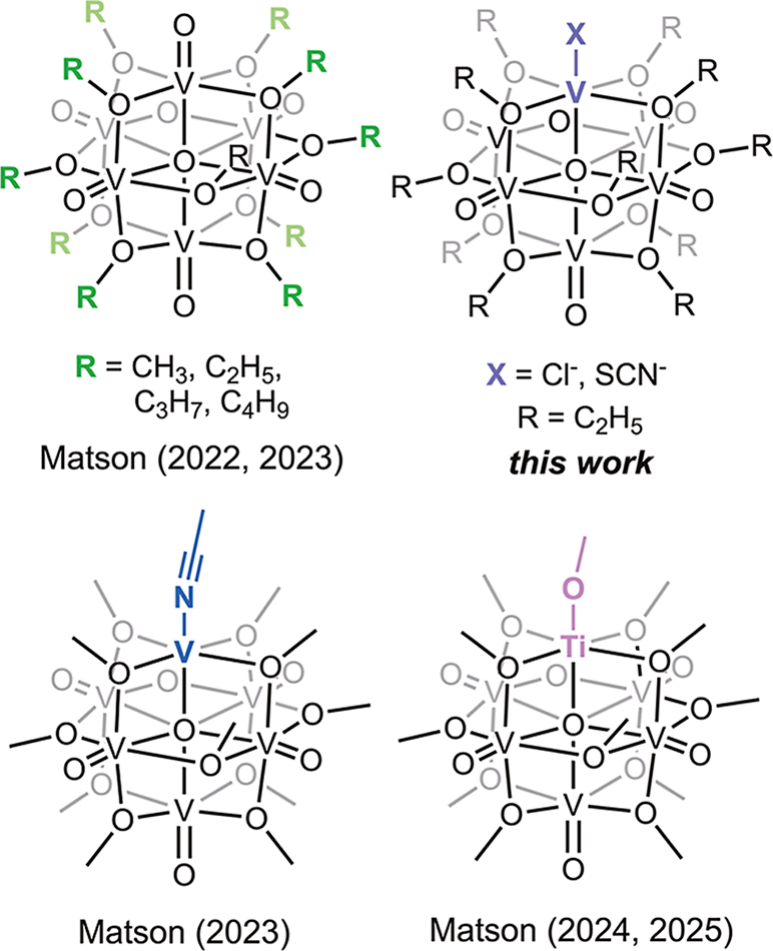
Examples of POV-alkoxides used by our group as models for PCET at pristine and doped MO_*x*_ surfaces.

Herein we describe the synthesis and characterization of a series of anion doped POV-ethoxides, TBA­[V_6_O_6_X­(OC_2_H_5_)_12_] (TBA = tetrabutylammonium; X = Cl, SCN) for the investigation of the impact of anionic dopants on PCET to the surface of these assemblies. We present these molecules as homogeneous models for understanding surface reactivity at halide-doped MO_*x*_ surfaces. Addition of an equivalent of 9,10-dihydrophenazine (H_2_Phen) to TBA­[V_6_O_6_X­(OC_2_H_5_)_12_] (X = Cl, SCN) results in the formation of the 2e^–^/2H^+^ reduced assemblies, TBA­[V_6_O_5_X­(OH_2_)­(OC_2_H_5_)_12_]. Notably, the BDFE for the chloride and thiocyanate derivatives are the same, and lower than that of TBA­[V_6_O_6_(OH_2_)­(OC_2_H_5_)_12_], but yield different degrees of rate enhancement. Collectively, this work provides insights into geometric and electronic consequences of anionic dopants, and the relationship between these characteristics and the kinetic parameters of PCET at the surface of POV-alkoxides.

## Experimental Section

### General Considerations

All manipulations were carried out in the absence of water and oxygen in a UniLab MBraun inert atmosphere glovebox under an atmosphere of dinitrogen. Glassware was oven-dried for a minimum of 4 h and cooled in an evacuated antechamber prior to use in the glovebox. Celite 545 (J. T. Baker) was dried in a Schlenk flask for at least 14 h at 150 °C under vacuum prior to use. All solvents were dried and deoxygenated on a Glass Contour System (Pure Process Technology, LLC) and stored over activated 3 Å molecular sieves (Fisher Scientific; sieves were activated at 150 °C under vacuum prior to use). Tetrabutylammonium chloride (TBACl), and tetrabutylammonium thiocyanate (TBASCN), and tetrabutylammonium hexafluorophosphate (TBAPF_6_) were purchased from Sigma-Aldrich and recrystallized 3 times from hot benzene (or ethanol for TBAPF_6_), dried under vacuum and brought into the glovebox, where the salts were stored over P_2_O_5_ to ensure they remain moisture-free over extended storage. Hydrazobenzene (H_2_Azo) was purchased from TCI Chemicals and used as received. [V_6_O_7_(OC_2_H_5_)_12_],^28^ [V_6_O_6_(OC_2_H_5_)_12_(MeCN)] (**V**_**6**_ **O**_**6**_^**0**^),^21^ 9,10-dihydrophenazine,^29^ and *d*_2_-9,10-dideuterophenazine^21^ were prepared according to previously reported methods.

^1^H NMR spectra were recorded on a Bruker DPX-500 MHz spectrometer locked on the signal of deuterated solvents. All chemical shifts were reported relative to the peak of residual ^1^H signal in the deuterated solvents. CD_3_CN and CDCl_3_ were purchased from Cambridge Isotope Laboratories, degassed by three freeze–pump–thaw cycles, and stored over activated 3 Å molecular sieves. Infrared (Fourier transform infrared, FT-IR; attenuated total reflection, ATR) spectra were recorded on a PerkinElmer Spectrum 3 Fourier transform infrared spectrophotometer and are reported in wavenumbers (cm^–1^). Electronic absorption measurements were recorded at room temperature in anhydrous dichloromethane in a sealed 1 cm quartz cuvette with an Agilent Cary 60 UV–vis spectrophotometer. Mass spectrometry analyses were performed on an Advion Expression^L^ compact mass spectrometer equipped with an electrospray probe and an ion-trap mass analyzer. Direct injection analysis was employed in all cases with a sample solution in acetonitrile. A single crystal of TBA­[V_6_O_6_SCN­(OC_2_H_5_)_12_] (**V**_**6**_ **O**_**6**_ **SCN** ^**–**^) was mounted on the tip of a thin glass optical fiber (goniometer heads) and mounted on a Bruker SMART APEX II CCD platform diffractometer for data collection at 100.0(5) K. The structure was solved using SHELXT-2014/5 and refined using SHELXL-2014/7.^30,31^ Elemental analyses were performed on a PerkinElmer 2400 Series II Analyzer at the CENTC Elemental Analysis Facility, University of Rochester.

Cyclic voltammetry experiments were carried out at room temperature in a nitrogen-filled glovebox using a Bio-Logic SP 150 potentiostat/galvanostat and the EC-lab software suite. Cyclic voltammograms (CVs) were recorded using a glassy carbon working electrode (*ø* = 3.0 mm) and a Pt wire auxiliary electrode, both purchased from CH Instruments, USA. A Ag^+/0^ nonaqueous reference electrode with 0.01 M AgNO_3_ and 0.10 M TBAPF_6_ in acetonitrile was purchased from Bio-Logic and used as the reference electrode for all cyclic voltammetry measurements. CVs were collected with 1 mM analyte in 0.10 M TBAPF_6_ acetonitrile solutions. All measurements were IR compensated at 85% with impedance taken at 100 kHz using the ZIR tool included with the EC-Lab software. All redox events were referenced against the ferrocene/ferrocenium (Fc^+/0^) redox couple.

#### Synthesis of TBA­[V_6_O_6_(OC_2_H_5_)_12_Cl], **V**_**6**_ **O**_**6**_ **Cl** ^**–**^

The synthetic procedure for the formation of **V**_**6**_ **O**_**6**_ **Cl** ^**–**^ was adapted from a previously reported method.^32^ In a 20 mL scintillation vial, [V_6_O_6_(OC_2_H_5_)_12_(MeCN)] (0.128 g, 0.130 mmol) was dissolved in 8 mL dichloromethane. TBACl (0.043 g, 0.156 mmol, 1.2 equiv) was weighed by difference and added to the solution as a solid. The reaction mixture was stirred at room temperature for 1 h. Subsequently, volatiles were removed under reduced pressure. The resulting brown solid was washed with diethyl ether (14 mL), and filtered over a bed of Celite (2.0 cm) on a medium-porosity frit. The brown solid was extracted with acetonitrile (4 mL) and filtered once more. The acetonitrile was removed under reduced pressure, affording the product, **V**_**6**_ **O**_**6**_ **Cl** ^**–**^, as a brown solid (0.149 g, 0.120 mmol, 92%). Characterization of **V**_**6**_ **O**_**6**_ **Cl** ^**–**^ matches that previously reported for the compound.^32^

#### Synthesis of TBA­[V_6_O_6_(OC_2_H_5_)_12_SCN], **V**_**6**_ **O**_**6**_ **SCN** ^**–**^

In a 20 mL scintillation vial, [V_6_O_6_(OC_2_H_5_)_12_(MeCN)] (0.045 g, 0.049 mmol) was dissolved in 8 mL dichloromethane. TBASCN (0.016 g, 0.054 mmol, 1.1 equiv) was weighed by difference and added as a solid to the solution. The reaction mixture was stirred at room temperature for 1 h. Subsequently, volatiles were removed under reduced pressure. The resulting brown solid was washed with a 1:1 mixture of pentane/diethyl ether (10 mL), then extracted with a 9:1 mixture of toluene/tetrahydrofuran (20 mL), and filtered over a bed of Celite (2.0 cm) on a medium-porosity frit. The solvent was removed under reduced pressure, and the brown solid was extracted with acetonitrile (4 mL) and filtered. The acetonitrile was removed under reduced pressure, affording **V**_**6**_ **O**_**6**_ **SCN** ^**–**^ as a brown solid (0.052 g, 0.042 mmol, 86%). ^1^H NMR (500 MHz, CDCl_3_): δ −21.93, −2.38, −0.45, 1.07 (TBA), 1.49 (TBA), 1.66 (TBA), 3.13 (TBA), 4.58, 22.11, 28.79 ppm. ESI-MS (−ve): *m*/*z* 1000 (100%, [V_6_O_6_(OC_2_H_5_)_12_SCN]^−^). FT-IR (ATR, cm^–1^): 2081 (SCN), 1043 (O_b_–C_2_H_5_), 953 (V=O_t_). UV–vis (CH_3_CN): 398 (ε = 2267), 1000 nm (ε = 322 M^–1^ cm^–1^). Elemental analysis for C_41_H_96_N_2_O_18_SV_6_ (MW = 1242.93 g/mol) calcd (%): C, 39.62; H, 7.79; N, 2.25. Found (%): C, 39.99; H, 7.68; N, 1.85.

#### Synthesis of TBA­[V_6_O_5_(OC_2_H_5_)_12_Cl­(MeCN)], **V**_**6**_ **O**_**5**_ **Cl** ^**–**^

In a 20 mL scintillation vial, **V**_**6**_ **O**_**6**_ **Cl** ^**–**^ (0.035 g, 0.029 mmol) was dissolved in 2 mL of acetonitrile. In a separate vial, 9,10-dihydrophenazine (0.006 g, 0.032 mmol, 1.1 equiv) was dissolved in acetonitrile (2 mL) and transferred to the solution of **V**_**6**_ **O**_**6**_ **Cl** ^**–**^. The reaction was stirred for 1 h, slowly changing from brown to red, after which volatiles were removed under reduced pressure. The resultant red residue was washed with diethyl ether (8 mL), filtered over a bed of Celite (2.0 cm), and the remaining red solid was extracted with acetonitrile (2 mL). The acetonitrile was removed under reduced pressure, yielding the product, **V**_**6**_ **O**_**5**_ **Cl** ^**–**^, as a pink powder (0.035 g, 0.028 mmol, 98%). ^1^H NMR (500 MHz, CD_3_CN): δ −41.44, −27.08, −19.42, −11.22, −7.99, −6.19, −3.22, −2.19, −1.49, 0.97 (TBA), 1.35 (TBA), 1.59 (TBA), 3.07 (TBA), 5.43, 6.12, 28.48, 30.8, 32.14, 33.42 ppm. FT-IR (ATR, cm^–1^): 1051 (O_b_–C_2_H_5_), 965 (V=O_t_). UV–vis (CH_3_CN): [λ, nm; (ε, M^–1^ cm^–1^)] 420 (535), 540 (390), 640 (245), 1000 (150). Elemental analysis for C_42_H_99_N_2_O_17_V_6_Cl·0.5Et_2_O (MW = 1282.41 g/mol) calcd (%): C, 41.21; H, 8.17; N, 2.18. Found (%): C, 41.7; H, 8.17; N, 2.14.

#### Synthesis of TBA­[V_6_O_5_(OC_2_H_5_)_12_SCN­(MeCN)], **V**_**6**_ **O**_**5**_ **SCN** ^**–**^

In a 20 mL scintillation vial, **V**_**6**_ **O**_**6**_ **SCN** ^**–**^ (0.010 g, 0.0079 mmol) was dissolved in 2 mL of acetonitrile. In a separate vial, 9,10-dihydrophenazine (0.0016 g, 0.0087 mmol, 1.1 equiv) was dissolved in acetonitrile (2 mL) and transferred to the solution of **V**_**6**_ **O**_**6**_ **SCN** ^**–**^. The reaction was stirred for 30 min, changing from brown to red, after which volatiles were removed under reduced pressure. The resultant red residue was washed with diethyl ether (14 mL), filtered over a bed of Celite (2.0 cm), and the remaining red solid was extracted with acetonitrile (2 mL). The acetonitrile was removed under reduced pressure, yielding the product, **V**_**6**_ **O**_**5**_ **SCN** ^**–**^, as a pink powder (0.008 g, 0.0063 mmol, 80%). ^1^H NMR (500 MHz, CD_3_CN): δ −35.95, −32.88, −30.79, −18.50, −17.28, −12.20, −9.88, −7.32, −3.15, −2.14, −1.51, −0.65, 0.97 (TBA), 1.35 (TBA), 1.59 (TBA), 3.07 (TBA), 4.06, 4.66, 30.76, 31.96, 33.29 ppm. FT-IR (ATR, cm^–1^): 2091 (SCN), 1051 (O_b_–C_2_H_5_), 954 (V=O_t_). UV–vis (CH_3_CN): [λ, nm; (ε, M^–1^ cm^–1^)] 420 (830), 530 (390), 630 (240), 1000 (240). Elemental analysis for C_43_H_99_N_3_O_17_SV_6_ calcd (%): C, 40.73; H, 7.87; N, 3.31. Found (%): C, 40.89; H, 7.56; N, 3.28.

### General Procedure for Determining Bond Dissociation Free Energies (BDFE­(O–H)_avg_)

*Method A.* In an N_2_-filled glovebox, separate J. Young tubes were charged with 300 μL of stock solution of POV-alkoxide in THF-*d*_8_ ([**V**_**6**_ **O**_**6**_ **Cl** ^**–**^] 16.4 mM; [**V**_**6**_ **O**_**6**_ **SCN** ^**–**^] 6.26 mM), and one equivalent of H_2_Azo in THF-*d*_8_ was added to each. The solution was diluted to 0.5 mL and the J. Young tube was capped and shaken several times to ensure homogeneity and allowed to sit at 21 °C (room temperature) for 5 days, whereupon the ^1^H NMR spectrum was collected. This procedure was repeated in triplicate. The extent of the reaction was determined through the relative concentrations of reduced H_2_Azo and oxidized azobenzene (Azo), under the assumption that H atom transfer occurs solely from the POV-alkoxide to substrate (i.e., for each reduced POV-alkoxide formed, we assume the oxidation of one molecule of hydrazobenzene). Calculating the BDFE­(O–H)­avg of the reduced POV-alkoxide in solution can then be performed through methods adapted from the Mayer^3^ group, using

1 $$BDFE{\left(E-H\right)}_{adj}=BDFE{\left(E-H\right)}_{avg}-\frac{1.364}{n}\;\log\left(\frac{\left[H_{2}E\right]}{\left[E\right]}\right)$$

where BDFE­(E–H)_adj_ is the adjusted BDFE of the organic substrate based on where the equilibrium of the system lies, BDFE­(E–H)_avg_ is the reported BDFE­(N–H)_avg_ of H_2_Azo (60.4 kcal mol^–1^ in THF), *n* is the number of H atoms transferred to one equivalent of POV-alkoxide (*n* = 2, as each assembly can accept two H atom equivalents), and [H_2_E] and [E] are the measured concentrations of reduced and oxidized versions of the respective substrate in solution at equilibrium.

*Method B.* Determination of BDFE­(O–H)_avg_ for each was also performed using reactions between each POV-alkoxide (**V**_**6**_ **O**_**6**_ **Cl** ^**–**^ or **V**_**6**_ **O**_**6**_ **SCN** ^**–**^) and 1–3 equiv of H_2_Azo. In an N_2_-filled glovebox, separate J. Young tubes were charged with a stock solution of POV-alkoxide in THF-*d*_8_ ([**V**_**6**_ **O**_**6**_ **Cl** ^**–**^] 23.6 mM, 21.2 μL; [**V**_**6**_ **O**_**6**_ **SCN** ^**–**^] 20.1 mM, 24.8 μL), and one equivalent of H_2_Azo (21.74 mM, 23 μL) in THF-*d*_8_ was added to each. The solution was diluted to 0.5 mL and the J. Young tube was capped and shaken several times to ensure homogeneity and allowed to sit at 21 °C (room temperature) for 1 day, whereupon the ^1^H NMR spectrum was collected. The J. Young tube was returned to the glovebox, and an additional 0.5 equiv of H_2_Azo (11.6 μL) was added. The sample was shaken several times to ensure homogeneity and allowed to sit at room temperature for 1 day. This procedure was repeated up to 3 equiv of H_2_Azo. The extent of the reaction was determined through the relative concentrations of reduced H_2_Azo and Azo, under the assumption that H atom transfer occurs solely from the POV-alkoxide to substrate (i.e., for each reduced POV-alkoxide formed, we assume the oxidation of one molecule of hydrazobenzene). Equation 1 was used to determine BDFE­(O–H)_avg_.

### General Procedure for Pseudo-first Order Rate Determination

Pseudo-first-order reaction conditions were used to establish the rate constant for PCET from H_2_Phen to each POV-alkoxide (**V**_**6**_ **O**_**6**_ **Cl** ^**–**^ or **V**_**6**_ **O**_**6**_ **SCN** ^**–**^).^33^ Reactions were prepared with a constant concentration of POV-alkoxide in MeCN ([**V**_**6**_ **O**_**6**_ **Cl** ^**–**^] = 0.5 mM; [V_6_O_6_SCN^–^] = 0.75 mM) in a long-neck quartz cuvette (1 cm path length) and sealed with a septa. Solutions were allowed to equilibrate to 25 °C before beginning monitoring at 1050 nm. After acquisition has begun, 10–22.4 equiv of H_2_Phen were injected through the rubber septa, with POV-alkoxide reduction monitored at 1050 nm via EAS. As the PCET reaction progressed, the absorbance decayed until the reaction reached completion, leveling to the absorbance for the respective O atom deficient species ([**V**_**6**_ **O**_**5**_ **Cl** ^**–**^] or [**V**_**6**_ **O**_**5**_ **SCN** ^**–**^]). The plots of absorbance over time were fit to the following equation by least-squares fitting (Figures S14 and S17).

2 $$A_{t}=A_{f}+\left(A_{i}-A_{f}\right)\;e^{-k_{obs}t}$$

where *A*_*t*_ is the calculated absorbance at time, *t*, in seconds, *A*_f_ is the absorbance value at the end of the experiment, *A*_i_ is the initial absorbance after injection of POV-alkoxide to the cuvette, and *k*_obs_ is the pseudo-first order rate constant. The excellent fit found for reaction curves indicated that the order of reductant in the rate expression was 1. Each experiment was repeated in triplicate. The slopes of the resultant *k*_obs_ vs [H_2_Phen] plots were normalized for the four (*n* = 4) possible reactive V^V^O sites on each POV-alkoxide, as well as the two possible H atoms which can be transferred from H_2_Phen, in order to determine the second order rate constant, *k*_PCET_ (M^–1^ s^–1^), such that

3 $$k_{PCET}=\frac{slope}{n_{V=O}\times 2_{H‐atoms}}$$

No induction period was observed in the pseudo-first order kinetics traces; as such, the *y*-intercept was held at the origin in all cases. The reported errors are the first significant figure of the difference between the determined slope and the confidence interval maximum. To determine the deuterium kinetic isotope effect (KIE), analogous pseudo-first order reactions were performed under identical conditions, using the deuterium-labeled reductant species 9,10-*d*_2_-dideuterophenazine (D_2_Phen) (Figures S20–S23). The prepared D_2_Phen used for these reactions was found to be 98% D-labeled using ^1^H NMR spectroscopy. Uncertainties associated with KIE was determined by accounting for 10% of the average value.

### General Procedure for Determining Activation Parameters

Eyring analysis was performed by collecting absorbance vs time data at 1050 nm with temperatures ranging between 15 and 45 °C. Reactions were assembled in an analogous fashion to the above kinetics experiments, with constant POV-alkoxide and reductant concentrations of 0.5 mM (**V**_**6**_ **O**_**6**_ **Cl** ^**–**^; 0.75 mM for **V**_**6**_ **O**_**6**_ **SCN** ^**–**^) and 7.4 mM (9.5 mM for **V**_**6**_ **O**_**6**_ **SCN** ^**–**^). Experiments were repeated in triplicate. Conversion of *k*_obs_ to *k*_PCET_ was done by dividing *k*_obs_ by the reductant concentration and number of sites available for PCET (4 equatorial vanadyls) and number of H atoms (2). Plotting ln­(*k*_PCET_/*T*) as a function of 1/*T* (temperature converted to K), the linear plot was used to solve for activation parameters using the below equations where *R* is the gas constant in units of cal/(mol K), *k*_Boltz_ is Boltzmann’s constant, and *h*_Planck_ is Planck’s constant. The activation parameters for the reduction of each POV-alkoxide are listed in Table 3 and Figures S15, S16, S18, and S19.

4 $$\ln\left(\frac{k_{PCET}}{T}\right)=-2273.5\times \frac{1}{T}+7.0695$$

5 $$\Delta H^{‡}=-2273.5\times R$$

6 $$\Delta S^{‡}=R\times \left[7.0695-\ln\left(\frac{k_{Boltz}}{h_{Planck}}\right)\right]$$

7 $$\Delta G^{‡}=\Delta H^{‡}-T\Delta S^{‡}$$

## Results and Discussion

### Synthesis of POV-Alkoxides with Anionic Dopants

Previously, our group has reported the synthesis and characterization of a Cl-doped POV-alkoxide, TEA­[V_6_O_6_Cl­(OC_2_H_5_)_12_] (TEA = tetraethylammonium).^32^ Spectroscopic analysis of this complex indicates the presence of a V­(V) site within the Lindqvist core, suggesting H atom uptake reactivity would be possible with this species. However, the reactivity of this POV-alkoxide with H atom transfer reagents proved difficult to study, as its solubility is limited in organic solvent (e.g., acetonitrile, MeCN). As such, a modified synthetic procedure was employed; addition of the longer chain countercation salt, tetrabutylammonium chloride (TBACl; Scheme 1) to V_6_O_6_(MeCN)­(OC_2_H_5_)_12_ (**V**_**6**_ **O**_**6**_^**0**^) results in the formation of the desired Cl-doped assembly, TBA­[V_6_O_6_Cl­(OC_2_H_5_)_12_] (**V**_**6**_ **O**_**6**_ **Cl** ^**–**^). Indeed, following workup, the characterization profile matches that for the previously reported Cl-substituted assembly. Notably, the use of a TBA countercation improves the isolated yield of **V**_**6**_ **O**_**6**_ **Cl** ^**–**^ (92%).

**Scheme 1:**
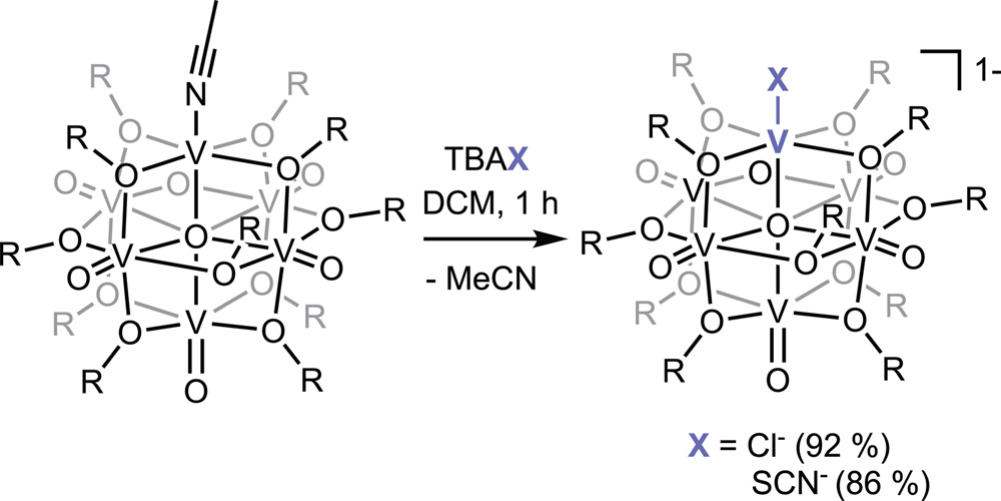
Synthesis of TBA­[V_6_O_6_X­(OC_2_H_5_)_12_] (X = Cl, SCN)

Interested in gaining a deeper understanding of the reactivity and coordination chemistry of the V^III^ site at the surface of **V**_**6**_ **O**_**6**_^**0**^, we turned our attention to generating the thiocyanate-bound conger of **V**_**6**_ **O**_**6**_ **Cl** ^**–**^. Specifically, this experiment was performed to understand how an ambidentate ligand would bind to the O atom vacancy site of **V**_**6**_ **O**_**6**_^**0**^ (e.g., nitrogen- or sulfur-binding; terminal vs bridging modes), as this information can provide insight into the substrate affinity of the V^III^ center (e.g., whether it prefers hard ligands like nitrogen or soft ligands like sulfur). The electronic and electrochemical effects of the coordination of these pseudohalides relative to the chloride derivative are also of interest.

The desired thiocyanate-substituted assembly, TBA­[V_6_O_6_SCN­(OC_2_H_5_)_12_] (**V**_**6**_ **O**_**6**_ **SCN** ^**–**^), is accessible through addition of tetrabutylammonium thiocyanate (TBASCN) to **V**_**6**_ **O**_**6**_^**0**^ in dichloromethane (Scheme 1, 86% yield). The ^1^H NMR spectrum of **V**_**6**_ **O**_**6**_ **SCN** ^**–**^ (−21.93, −2.38, −0.45, 4.58, 22.11, 28.79 ppm) exhibits the expected six-peak pattern, wherein the chemical shifts resemble signals of the bridging ethoxide moieties observed in the ^1^H NMR spectrum of **V**_**6**_ **O**_**6**_ **Cl** ^**–**^ (Figure 2). These resonances are significantly shifted from the starting material, **V**_**6**_ **O**_**6**_^**0**^ (δ = 35.61, 17.13, 2.73, 0.19, −3.17, −23.99 ppm; Figure 2). The chemical composition and purity of **V**_**6**_ **O**_**6**_ **SCN** ^**–**^ is confirmed by ESI-MS (Figure S1; [V_6_O_6_(OC_2_H_5_)_12_SCN]^−^, *m*/*z* = 1000) and combustion analysis (see Experimental Section for details).

**Figure 2:**
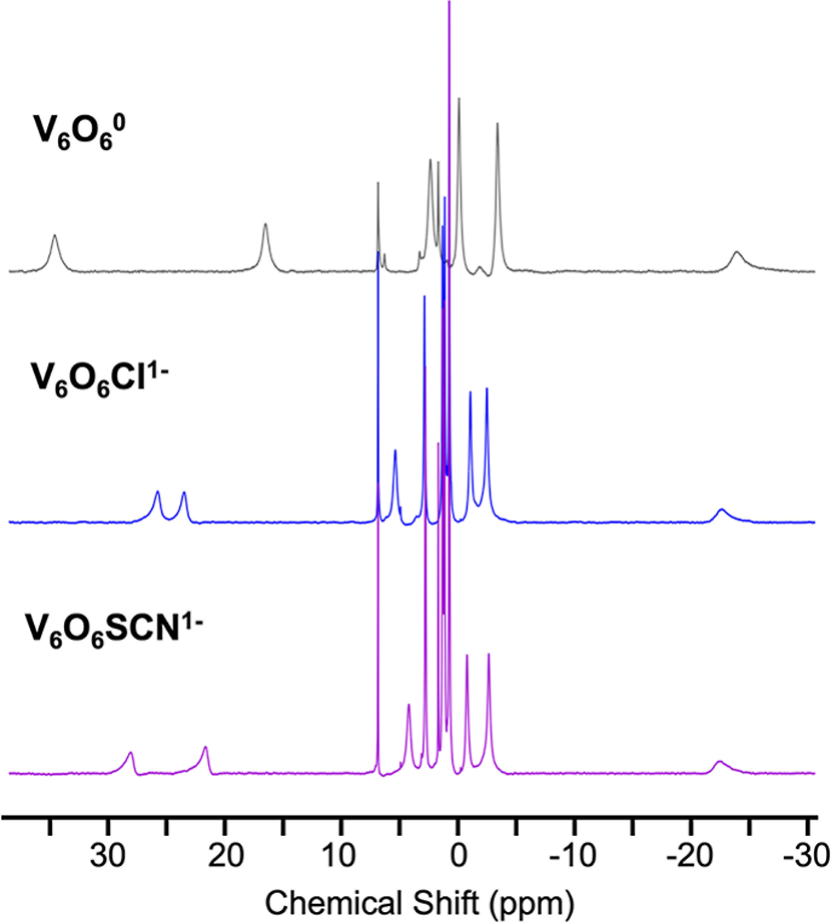
^1^H NMR spectra of **V**_**6**_ **O**_**6**_^**0**^ (gray, top), **V**_**6**_ **O**_**6**_ **Cl** ^**–**^ (blue, middle), **V**_**6**_ **O**_**6**_ **SCN** ^**–**^ (purple, bottom) collected in MeCN-*d*_3_ at 21 °C.

Our group and others have demonstrated that the electronic structure of POV-alkoxides can be interrogated using electronic absorption and infrared (IR) spectroscopies (Figure 3).^20,22,23,28,34^ The electronic absorption spectrum (EAS) of **V**_**6**_ **O**_**6**_ **SCN** ^**–**^ (UV–vis/near-infrared) contains an intervalence charge transfer (IVCT) transition expected for mixed-valent V^IV^/V^V^ systems at ∼1000 nm (ε = 322 M^–1^ cm^–1^), along with a transition assigned to IVCT mixed with ligand-to-metal charge-transfer (LMCT) at 398 nm (ε = 2267 M^–1^ cm^–1^; Figure 3a, Table 1). The overall spectrum of **V**_**6**_ **O**_**6**_ **SCN** ^**–**^ resembles that reported for **V**_**6**_ **O**_**6**_ **Cl** ^**–**^ (398 nm, ε = 3710 M^–1^ cm^–1^; 1000 nm, ε = 478 M^–1^ cm^–1^), consistent with an analogous electronic structure and oxidation state distribution of vanadium centers (V^III^V^IV^_4_V^V^).^32,35^ The IR spectrum of **V**_**6**_ **O**_**6**_ **SCN** ^**–**^ possesses ν­(O_b_–C_2_H_5_) (1043 cm^–1^) and ν­(V=O_t_) (953 cm^–1^) vibrations with Δν of ∼90 cm^–1^ (Figures 3b and S2, Table 1). The Δν value is similar to **V**_**6**_ **O**_**6**_ **Cl** ^**–**^ (Δν = 84 cm^–1^), providing additional support for identical oxidation state distributions of vanadium ions within these assemblies.^28^ A diagnostic vibration of the thiocyanate ligand was also observed (Figure 3, Table 1). The ν­(SCN) of **V**_**6**_ **O**_**6**_ **SCN** ^**–**^ (2081 cm^–1^) exhibits a modest shift from free TBASCN (2064 cm^–1^). The ν­(SCN) value is similar to other thiocyanate (2058–2085 cm^–1^) V^III^ complexes reported in the literature.^36,37^ This result supports the direct coordination of the anion to the POV-alkoxide, most likely through the open coordination site of the oxygen-deficient vanadium center at the surface of the assembly.

**Figure 3:**
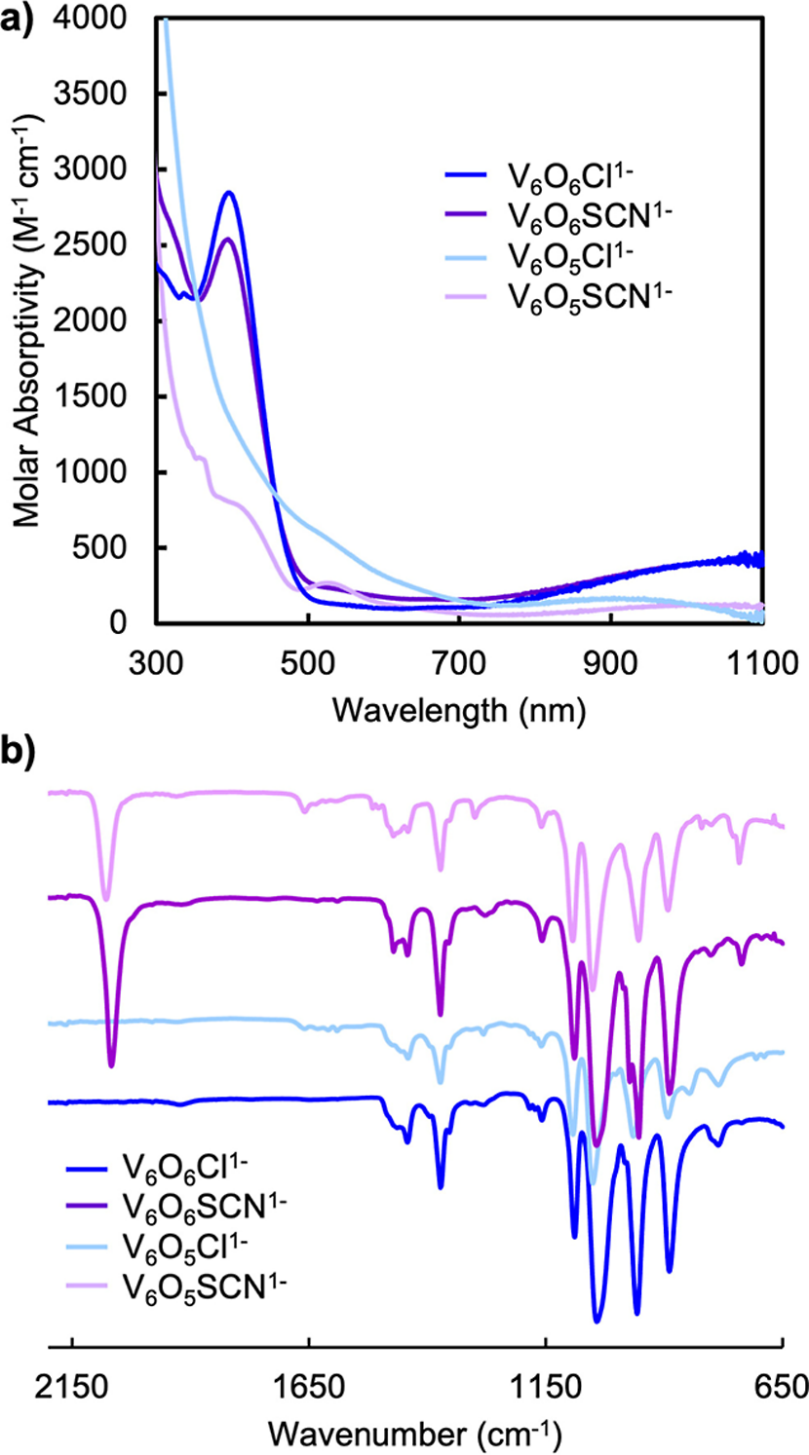
(a) Electronic absorption spectra collected in MeCN at 25 °C and (b) IR spectra of **V**_**6**_ **O**_**6**_ **Cl** ^**–**^ (blue), **V**_**6**_ **O**_**6**_ **SCN** ^**–**^ (purple), **V**_**6**_ **O**_**5**_ **Cl** ^**–**^ (light blue), **V**_**6**_ **O**_**5**_ **SCN** ^**–**^ (light purple).

**Table 1:** Infrared and Electronic Absorption Parameters for **V**_**6**_ **O**_**6**_^**0**^, **V**_**6**_ **O**_**6**_ **Cl** ^**–**^, **V**_**6**_ **O**_**6**_ **SCN** ^**–**^, and **V**_**6**_ **O**_**7**_^**–**^

species (ox. state distribution)	ν(SCN) (cm^–1^)	ν(V=O_t_) (cm^–1^)	ν(O_b_–R) (cm^–1^)	Δν (cm^–1^)	λ (nm) (ε (M^–1^ cm^–1^))
**V**_**6**_ **O**_**6**_^**0**^ (V^III^V^IV^_4_V^V^)		964	1040	76	392 (2740), 1000 (407)
**V**_**6**_ **O**_**6**_ **Cl** ^**–**^ (V^III^V^IV^_4_V^V^)		956	1040	84	398 (2807), 1000 (400)
**V**_**6**_ **O**_**6**_ **SCN** ^**–**^ (V^III^V^IV^_4_V^V^)	2081	953	1043	90	398 (2267), 1000 (322)
**V**_**6**_ **O**_**7**_^**–**^ (V^IV^_5_V^V^)		945	1045	100	390 (5750), 1000 (1331)

To unambiguously confirm formation and binding mode of the thiocyanate complex, single crystals suitable for X-ray diffraction experiments were analyzed. Crystals were grown from slow diffusion of pentane into a concentrated solution of **V**_**6**_ **O**_**6**_ **SCN** ^**–**^ in 2-methyltetrahydrofuran. Refinement of data revealed a single POV-alkoxide within the unit cell with all atoms located in general positions (Figure 4). The thiocyanate ligand is bound through nitrogen, revealing a preference for the “hard” binding site of nitrogen over sulfur at V­(III). The generated V–N–C angle of 177.8(2)° is consistent with most N-bound thiocyanate–metal complexes.^37^ Bond valence sum calculations confirm the anionic dopant is bound to a V­(III) center, with an oxidized vanadium ion, V­(V), located within the equatorial plane (Table S2).

**Figure 4:**
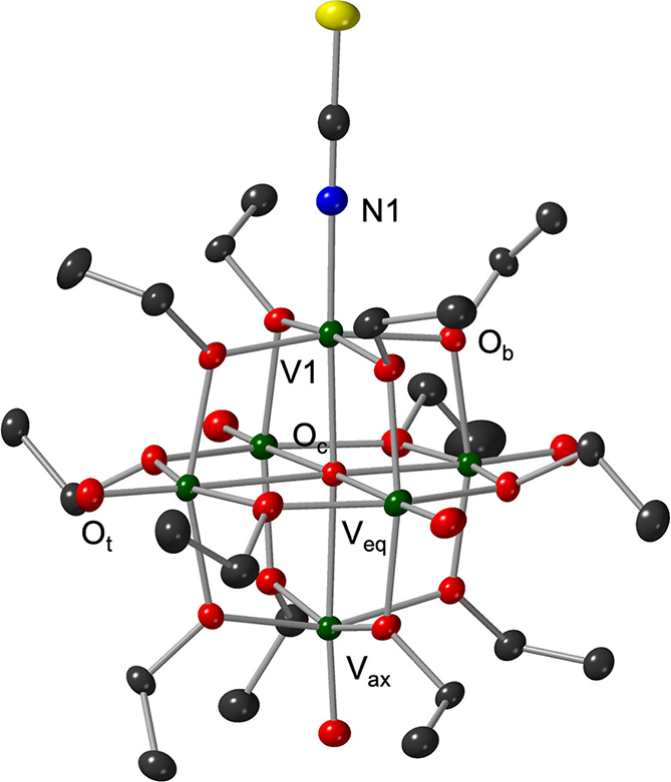
Molecular structure of **V**_**6**_ **O**_**6**_ **SCN** ^**–**^ shown with 50% probability thermal ellipsoids. Counterion molecules and hydrogens have been omitted for clarity. Crystallographic parameters are summarized in Tables 2 and S1. Colors: C, gray; O, red; V, green; N, blue; S, yellow.

To understand the impact of the (pseudo)­halide dopants on the structural metrics of the Lindqvist core, vanadium–oxygen bond lengths were compared against core bond lengths and angles in previously reported structures of POV-alkoxides (Table 2). Due to disorder in the crystal structure of the Cl-doped assembly, **V**_**6**_ **O**_**6**_ **Cl** ^**–**^, direct comparisons of bond metrics cannot be performed.^32^ The impacts of anionic ligand binding to the core structure were compared against POV-methoxides previously reported by our group; we note that **V**_**6**_ **O**_**6**_ **SCN** ^**–**^ has 12 bridging-ethoxide ligands, however we do not expect that the length of these alkoxide moieties will have a substantial impact on the structure of the Lindqvist core.^22,35^ In the case of **V**_**6**_ **O**_**6**_ **SCN** ^**–**^, the defect site (V1) yields a shorter bond to the central oxo (O_c_), 2.130(4) Å, compared to the average central oxo bond length reported for [V_6_O_7_(OCH_3_)_12_]^−^ (2.311 Å), while the O_c_ bond trans to the dopant site is elongated to 2.330(4) Å.^21^ Additionally, a near-square angle with the equatorial plane is introduced (V_*x*_–O_c_–V_eq_ = 90.3°), introducing strain which may activate vanadyl groups positioned in the equatorial plane of the compound (vide infra). The observed structural perturbations in **V**_**6**_ **O**_**6**_ **SCN** ^**–**^ as compared to the parent POV-alkoxide resemble changes in V–O lattice distances of O atom deficient assemblies (e.g., shortened V_*x*_–O_c_ bond, contracted V_*x*_–O_c_–V_eq_ bond angles). In all cases, short V–O distances are observed between the central oxido ligand and the vanadium center bearing a defect site (2.07–2.13 Å). It is worth noting that the coordination of the thiocyanate ion to the reduced vanadium center appears to alleviate some of the geometric strain imposed by an O atom vacancy (V_*x*_–O_c_–V_eq_ (avg) = 90.3°, V_*x*_–O_c_ = 2.068(4) Å).^21^

**Table 2:** Pertinent Bond Metrics of **V**_**6**_ **O**_**6**_ **SCN** ^**–**^, [V_6_O_7_(OCH_3_)_12_]^−^, and [V_6_O_6_(OCH_3_)_12_(MeCN)]^−^ ^a^

bond or angle	**V**_**6**_ **O**_**6**_ **SCN** ^**–**^	^b^[V_6_O_7_(OCH_3_)_12_]^−^	^c^[V_6_O_6_(OCH_3_)_12_(MeCN)]^−^
V–O_t_ (avg, Å)	1.601	1.606	1.600
V_eq_–O_c(avg)_ (Å)	2.320	2.311	2.326
V_ax_–O_c_ (Å)	2.330(4)	--	2.354(4)
V1–O_c_ (Å)	2.130(4)	--	2.068(4)
V1–O_c_–V_eq_ (avg)	90.3	109.9	89.55

a V1, defect V site; V_ax_, axial V; V_eq_, equatorial V; O_c_, central oxo; O_t_, terminal oxo.

b Values from structures previously reported.^38^

c Values from previously reported structure.^21^

To study the influence of ligand substitution on the electrochemical properties of the POV-alkoxide, the cyclic voltammogram (CV) of **V**_**6**_ **O**_**6**_ **SCN** ^**–**^ was collected in dichloromethane (Figure 5, Table 3). **V**_**6**_ **O**_**6**_ **SCN** ^**–**^ exhibits four quasi-reversible redox events (*E*_1/2_ = −0.99, −0.43, +0.29, +0.96 V vs Fc^+/0^). The open circuit potential (OCP) of **V**_**6**_ **O**_**6**_ **SCN** ^**–**^ is measured at −0.72 V vs Fc^0/+^, placing the [V^III^V^IV^_4_V^V^] oxidation state distribution between the two most reducing redox events. Interestingly, the CV of **V**_**6**_ **O**_**6**_ **SCN** ^**–**^ is almost superimposable with that of **V**_**6**_ **O**_**6**_ **Cl** ^**–**^. The similar electrochemical behavior displayed by the pseudohalide- and chloride-functionalized POV-alkoxides is not surprising, considering that thiocyanate anions have similar charge and donor–acceptor capabilities as chloride ligands. These events are shifted oxidatively relative to that of **V**_**6**_ **O**_**7**_^**0**^ and reductively relative to **V**_**6**_ **O**_**6**_^**0**^, suggesting the addition of a formal anionic dopant imposes less impact on the redox chemistry than formal vacancy formation.^32^ These changes are attributed to the addition of a formal charge in the case of **V**_**6**_ **O**_**6**_ **Cl** ^**–**^ and **V**_**6**_ **O**_**6**_ **SCN** ^**–**^. While O-atom site defects have been treated as a type of anionic dopant in MO_*x*_, by way of the 2 e^–^ pair left upon cleavage of the M=O bond, a formal charge is not generated upon removal of an oxygen atom. However, as observed in **V**_**6**_ **O**_**6**_ **Cl** ^**–**^, as well as **V**_**6**_ **O**_**6**_ **SCN** ^**–**^, while the electronic distribution across the POV-alkoxide is the same as **V**_**6**_ **O**_**6**_^**0**^ (i.e., V^III^V^IV^_4_V^V^), the assembly gains an overall negative charge, which reduces its oxidizing potential.

**Figure 5:**
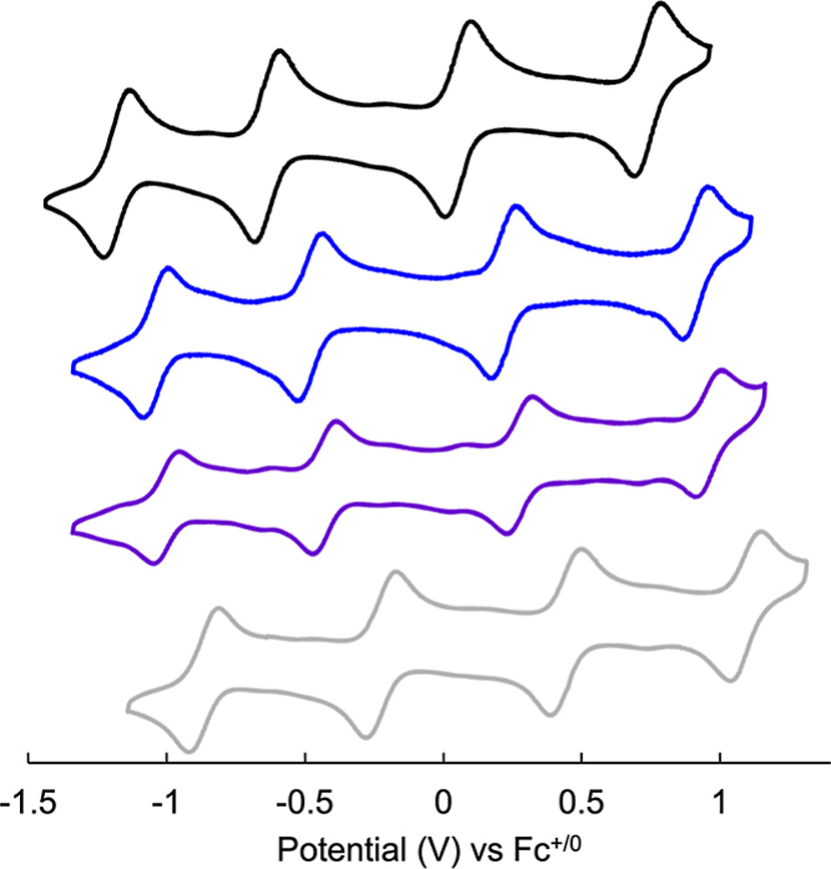
Cyclic voltammograms of **V**_**6**_ **O**_**7**_^**0**^ (black), **V**_**6**_ **O**_**6**_ **Cl** ^**–**^ (blue), **V**_**6**_ **O**_**6**_ **SCN** ^**–**^ (purple), and **V**_**6**_ **O**_**6**_^**0**^ (gray) collected in dichloromethane, with 0.1 M TBAPF_6_ as supporting electrolyte, at a scan rate of 500 mV/s, at 21 C.

**Table 3:** Electrochemical Parameters of **V**_**6**_ **O**_**7**_^**0**^, **V**_**6**_ **O**_**6**_ **Cl** ^**–**^, **V**_**6**_ **O**_**6**_ **SCN** ^**–**^, and **V**_**6**_ **O**_**6**_^**0**^ in Dichloromethane

redox couple	**V**_**6**_ **O**_**7**_^**0**^ *E*_1/2_ (vs Fc^+/0^)	redox couple	**V**_**6**_ **O**_**6**_^**0**^ *E*_1/2_ (vs Fc^+/0^)	**V**_**6**_ **O**_**6**_ **Cl** ^**–**^ *E*_1/2_ (vs Fc^+/0^)	**V**_**6**_ **O**_**6**_ **SCN** ^**–**^ *E*_1/2_ (vs Fc^+/0^)
[V^IV^_6_]/[V^IV^_5_V^V^]	–1.16	[V^III^V^IV^_5_]/[V^III^V^IV^_4_V^V^]	–0.85	–1.02	–0.99
[V^IV^_5_V^V^]/[V^IV^_4_V^V^_2_]	–0.62	[V^III^V^IV^_4_V^V^]/[V^III^V^IV^_3_V^V^_2_]	–0.21	–0.47	–0.43
[V^IV^_4_V^V^_2_]/[V^IV^_3_V^V^_3_]	+0.07	[V^III^V^IV^_3_V^V^_2_]/[V^III^V^IV^_2_V^V^_3_]	+0.46	+0.23	+0.29
[V^IV^_3_V^V^_3_]/[V^IV^_2_V^V^_4_]	+0.75	[V^III^V^IV^_2_V^V^_3_]/[V^III^V^IV^V^V^_4_]	+1.11	+0.92	+0.96

### H Atom Uptake at **V**_**6**_ **O**_**6**_ **X** ^**–**^

Introduction of site-defects in extended materials has been proposed to activate adjacent metal-oxo sites to the defect for more facile H atom uptake.^8^ Indeed, in both O-atom deficient and Ti­(IV)-substituted POV-alkoxides, introduction of a substitutional dopant yields an increased rate in H atom uptake in the equatorial plane of the POV-alkoxide, *cis*- to the defect site.^24–26^ In each of these cases the geometric strain introduced by the defect results in the activation of the equatorial vanadyl sites, localizing H atom uptake *cis*- to the defect. Interested in extending our understanding of the role of coordinating anions in H atom uptake at the surface of metal oxides, we investigated the reactivity of **V**_**6**_ **O**_**6**_ **Cl** ^**–**^ and **V**_**6**_ **O**_**6**_ **SCN** ^**–**^ with 9,10-dihydrophenazine (H_2_Phen). We note that our original intent was to compare the reactivity observed for the (pseudo)­halide substituted assemblies with an oxygen-deficient species (i.e., H atom uptake at **V**_**6**_ **O**_**6**_^**0**^); however, attempts to generate the divacant assembly, V_6_O_5_(OC_2_H_5_)_12_, from **V**_**6**_ **O**_**6**_^**0**^ resulted in formation of a pink product with poor solubility in MeCN, obscuring comparative kinetic investigations.

Addition of an equivalent of H_2_Phen (BDFE­(N–H)_avg_ = 59.2 kcal mol^–1^ in MeCN) to **V**_**6**_ **O**_**6**_ **Cl** ^**–**^ results in a color change from brown to red over the course of ∼2 h (Scheme 2, Figure S3). Analysis of the crude reaction mixture via ^1^H NMR spectroscopy reveals formation of phenazine (Phen, 7.92 and 8.24 ppm), as well as 16 paramagnetically shifted and broadened resonances attributed to 8 unique bridging ethoxide sites of [TBA]­[V_6_O_5_Cl­(MeCN)­(OC_2_H_5_)_12_] (**V**_**6**_ **O**_**5**_ **Cl** ^**–**^) (Figure 6). This is consistent with a reduction in the symmetry from *C*_4*v*_ in the parent POV-alkoxide, **V**_**6**_ **O**_**6**_ **Cl** ^**–**^, to *C*_*s*_ symmetry upon vacancy formation at a vanadyl site *cis*- to that of the Cl dopant.

**Scheme 2:**
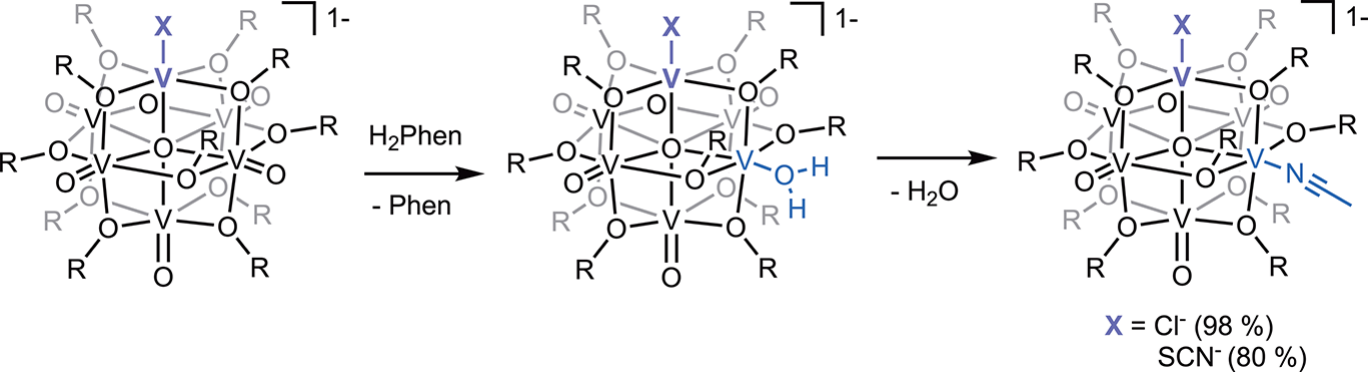
H Atom Uptake at the Surface of X-Substituted POV-Alkoxides^a^

a Synthesis of TBA­[V_6_O_5_X­(MeCN)­(OC_2_H_5_)_12_] (X = Cl, **V**_**6**_ **O**_**5**_ **Cl** ^**–**^; SCN, **V**_**6**_ **O**_**5**_ **SCN** ^**–**^).

**Figure 6:**
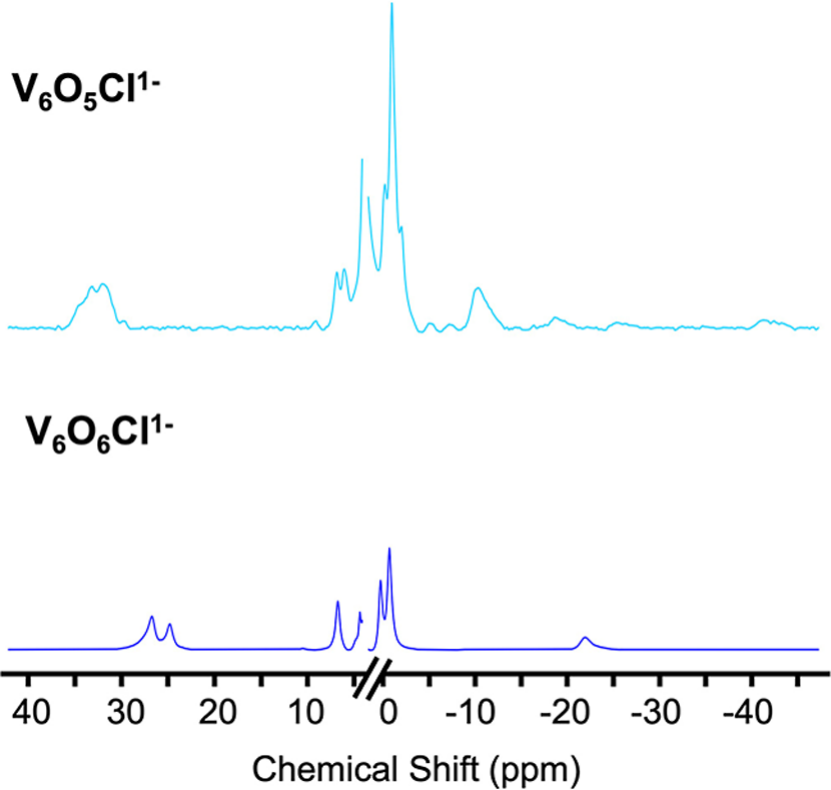
^1^H NMR spectrum of **V**_**6**_ **O**_**5**_ **Cl** ^**–**^ (light blue, top) and **V**_**6**_ **O**_**6**_ **Cl** ^**–**^ (blue, bottom), omitting region for TBA 1–4 ppm, in MeCN-*d*_3_ at 21 °C.

Further evidence for vanadyl reduction at the surface of **V**_**6**_ **O**_**6**_ **Cl** ^**–**^ upon addition of H_2_Phen is observed in both the electronic absorption and infrared spectra of the product (Figure 3). EAS of **V**_**6**_ **O**_**5**_ **Cl** ^**–**^ reveals the loss of two features that are attributed to the presence of the V­(V) center in the Lindqvist core: (1) a band at 398 nm corresponding to the LMCT from an oxo to a V­(V) site, and (2) the IVCT band attributed to the charge transfer from a V­(IV) to V­(V). Along with the loss of these features, three new, weak transitions are observed at 420 (535 M^–1^ cm^–1^), 540 (390 M^–1^ cm^–1^), and 640 (245 M^–1^ cm^–1^) nm, which are attributed to d–d transitions in the V­(IV) sites (Figure 3a). Additionally, the IR spectra of **V**_**6**_ **O**_**5**_ **Cl** ^**–**^ supports reduction of the assembly (Figures 3b and S4). Whereas **V**_**6**_ **O**_**6**_ **Cl** ^**–**^ possesses an energy gap of 86 cm^–1^ between the ν­(O_b_–R) (1051 cm^–1^) and ν­(V=O_t_) (965 cm^–1^) features, these transitions are shifted further apart from one another in the case of **V**_**6**_ **O**_**5**_ **Cl** ^**–**^ (ν­(O_b_–R) 1055 cm^–1^, ν­(V=O_t_) 956 cm^–1^; Δν = 99 cm^–1^). We note that similar changes in values of Δν have been observed upon O atom defect formation in POV-alkoxides previously by our group.^20,21,25,39^ Collectively, these changes in the electronic absorption and infrared spectra are consistent with H atom uptake at the V­(V) center to form a V­(III)-aquo moiety.

Next, we extended our investigations of H atom uptake reactivity at the surface of anion-doped POV-alkoxides to the thiocyanate-substituted assembly. Upon addition of an equivalent of H_2_Phen to **V**_**6**_ **O**_**6**_ **SCN** ^**–**^, a color change from brown to red occurs within 30 min (Figure S5); we note that this reaction time is much shorter than what was qualitatively observed in the case of Cl-substituted assembly (∼2 h). Analysis of the crude reaction mixture by ^1^H NMR spectroscopy reveals the expected formation of Phen, and a set of paramagnetically shifted and broadened resonances for the POV-alkoxide product that are quite similar to those described above for **V**_**6**_ **O**_**5**_ **Cl** ^**–**^ (Figure S6). Further evidence for reduction of the POV-alkoxide is observed in the electronic absorption spectrum of the product of H atom uptake (420 nm, ε = 830 M^–1^ cm^–1^; 530 nm, ε = 390 M^–1^ cm^–1^; 630 nm, ε = 240 M^–1^ cm^–1^; Figure 3a). The IR spectrum also reveals similar changes to the energy gap of ν­(O_b_–R) and ν­(V=O_t_) transitions of the thiocyanate-substituted POV-alkoxide upon reduction (Δν = 90 → 97 cm^–1^; Figures 3b and S7). Retention of the anionic dopant is confirmed by FT-IR spectroscopy, wherein the ν­(SCN) shifts slightly up in energy, by 3 cm^–1^. Minimal shifting suggests the ligand is not significantly impacted by reduction of adjacent vanadyl sites. Collectively, these data support successful formation of the O atom deficient POV-alkoxide, [TBA]­[V_6_O_5_SCN­(MeCN)­(OC_2_H_5_)_12_] (**V**_**6**_ **O**_**5**_ **SCN** ^**–**^).

The products of H atom uptake, **V**_**6**_ **O**_**5**_ **Cl** ^**–**^ and **V**_**6**_ **O**_**5**_ **SCN** ^**–**^, were further characterized by cyclic voltammetry. Three redox couples are observed for each reduced species (**V**_**6**_ **O**_**5**_ **Cl** ^**–**^, *E*_1/2_ = −0.364, 0.186, and 0.761 V; **V**_**6**_ **O**_**5**_ **SCN** ^**–**^ *E*_1/2_ = −0.461, 0.093, and 0.667 V vs Fc^+/0^; Figures S8 and S9). In both experiments, the OCP of the POV-alkoxide is found at potentials lower than the most reducing redox couple (**V**_**6**_ **O**_**5**_ **Cl** ^**–**^, OCP = −0.371 V; **V**_**6**_ **O**_**5**_ **SCN** ^**–**^, OCP = −0.641 V), suggesting the assemblies are in their lowest redox state. Notably, unlike their oxidized congeners, the O atom deficient chloride and thiocyanate complexes reveal strikingly different electrochemical profiles, where **V**_**6**_ **O**_**5**_ **SCN** ^**–**^ is shifted to more reducing potentials than **V**_**6**_ **O**_**5**_ **Cl** ^**–**^. This would suggest that in the reduced assembly, the thiocyanate provides more electron density to the core, acting as a stronger π-donor ligand, and providing more stability to the reduced form of the POV-alkoxide. The π-accepting and π-donating character of the thiocyanate ligand may allow for attenuated binding, where the donating and accepting character compensates for changes to the charge of the complex.

To understand the effects of the identity of the (pseudo)­halide dopant on the thermodynamics of H atom uptake at POV-alkoxides, we quantified the bond dissociation free energy (BDFE­(O–H)_avg_) of the aquo unit formed at the surface of the reduced assembly. Previous work from our group has established the first H atom transfer to be the rate-determining step, with the second H atom transfer being rapid and irreversible, resulting in an average BDFE­(O–H) for both H atoms.^21,40^ The BDFE­(E–H) provides insight into the driving force for H atom uptake, or release of a radical hydrogen, which allows for comparison of the thermodynamic implications of defects at the surface of POV-alkoxides. To obtain a BDFE­(O–H)_avg_, we employed the equilibrium methods described by Mayer.^3^ Having observed full conversion to the vacancy product upon stoichiometric addition of H_2_Phen (BDFE­(N–H)_avg_ = 59.2 kcal mol^–1^ in MeCN), we turned to a substrate with a higher BDFE­(N–H)_avg_, hydrazobenzene (H_2_Azo) at 60.4 kcal mol^–1^ in THF. Stoichiometric **V**_**6**_ **O**_**6**_ **Cl** ^**–**^ and H_2_Azo were combined in THF-*d*_8_ and allowed to equilibrate for 5 days. Analysis of the relative concentrations of reduced and oxidized substrate reveals a modest BDFE­(O–H)_avg_ of 59.4 ± 0.4 kcal mol^–1^ for the reduced and protonated POV-alkoxide (Figures S10 and S11, Table S3). Under similar conditions, the equilibrium established between **V**_**6**_ **O**_**6**_ **SCN** ^**–**^ and H_2_Azo also reveals a BDFE­(O–H)_avg_ of 59.4 ± 0.4 kcal mol^–1^ for the reduced and protonated POV-alkoxide (Figures S12 and S13, Table S4). We note that the BDFE­(O–H)_avg_ values for **V**_**6**_ **O**_**5**_ **Cl** ^**–**^ and **V**_**6**_ **O**_**5**_ **SCN** ^**–**^ are equivalent, suggesting that the identity of anionic dopant does not influence the thermodynamics.

Despite identical thermodynamic driving forces for H atom uptake in **V**_**6**_ **O**_**6**_ **Cl** ^**–**^ and **V**_**6**_ **O**_**6**_ **SCN** ^**–**^, the two assemblies exhibit disparate apparent rates of reaction. Similar phenomena has been observed previously in the POV-methoxide system. An O atom defect generates a weaker BDFE­(O–H)_avg_ (60.7 kcal mol^–1^, V_6_O_6_(OCH_3_)_12_; 62.3 kcal mol^–1^, V_6_O_7_(OCH_3_)_12_) than the fully oxygenated POV-methoxide, yet kinetically enhances H atom uptake by 100-fold.^24^ In this case, the rate increase is attributed to relieving the strain on the lattice imposed by the local V­(III) site. The addition of the anionic dopant similarly yields a localized V­(III) site, although the impact of these structural perturbations are expected to be consistent for the Cl- and SCN-doped assemblies. Alternatively, a mechanistic change may similarly account for the enhancement of the reaction rate, as is observed in Ti-doped POV-alkoxides.^25^ As such, our attention shifted to kinetic studies to better understand the observed differences in rates of reduction.

Initial experiments focused on the elucidation of the rate expression for H atom transfer from H_2_Phen to **V**_**6**_ **O**_**6**_ **Cl** ^**–**^. Kinetic analyses of PCET (*k*_PCET_) from substrate to POV-alkoxide was achieved by monitoring reaction progress using EAS under pseudo-first order reaction conditions (excess H_2_Phen). The extent of reaction progress was assessed by monitoring the loss of the IVCT band at 1050 nm in MeCN. This band has no overlap with organic product signals, providing a handle to directly observe POV-alkoxide reduction upon H atom uptake at the surface of the assembly. The pseudo-first order rate constant (*k*_obs_) is obtained from the fitting of an exponential decay function of the loss in absorbance using the least-squares method (see Experimental Section for more details, Figure S14). The plot of the obtained *k*_obs_ versus the concentration of reductant provides a linear correlation indicating an overall second-order rate constant for the reaction (Figure 7). To account for the four distinct sites possible for uptake of 2 H atoms (vanadyl reduction occurs exclusively at a cis-position in **V**_**6**_ **O**_**6**_ **Cl** ^**–**^, and there are four equatorial vanadyl positions), the second order rate constant is corrected by a factor of 8 to reveal a *k*_PCET_ value of 0.0545 ± 0.005 M^–1^ s^–1^. Interestingly, the rate of H atom uptake at the surface of the Cl-doped assembly exhibits a 2-fold rate enhancement in comparison to **V**_**6**_ **O**_**7**_^**–**^ (0.020 ± 0.002 M^–1^ s^–1^, Table 4), despite similar driving forces for H atom uptake across the two assemblies (BDFE­(O–H)_avg_ **V**_**6**_ **O**_**6**_ **Cl** ^**–**^ = 59.4 ± 0.4 kcal mol^–1^; BDFE­(O–H)_avg_ **V**_**6**_ **O**_**7**_^**–**^ = 59.7 ± 0.1 kcal mol^–1^).^22^

**Figure 7:**
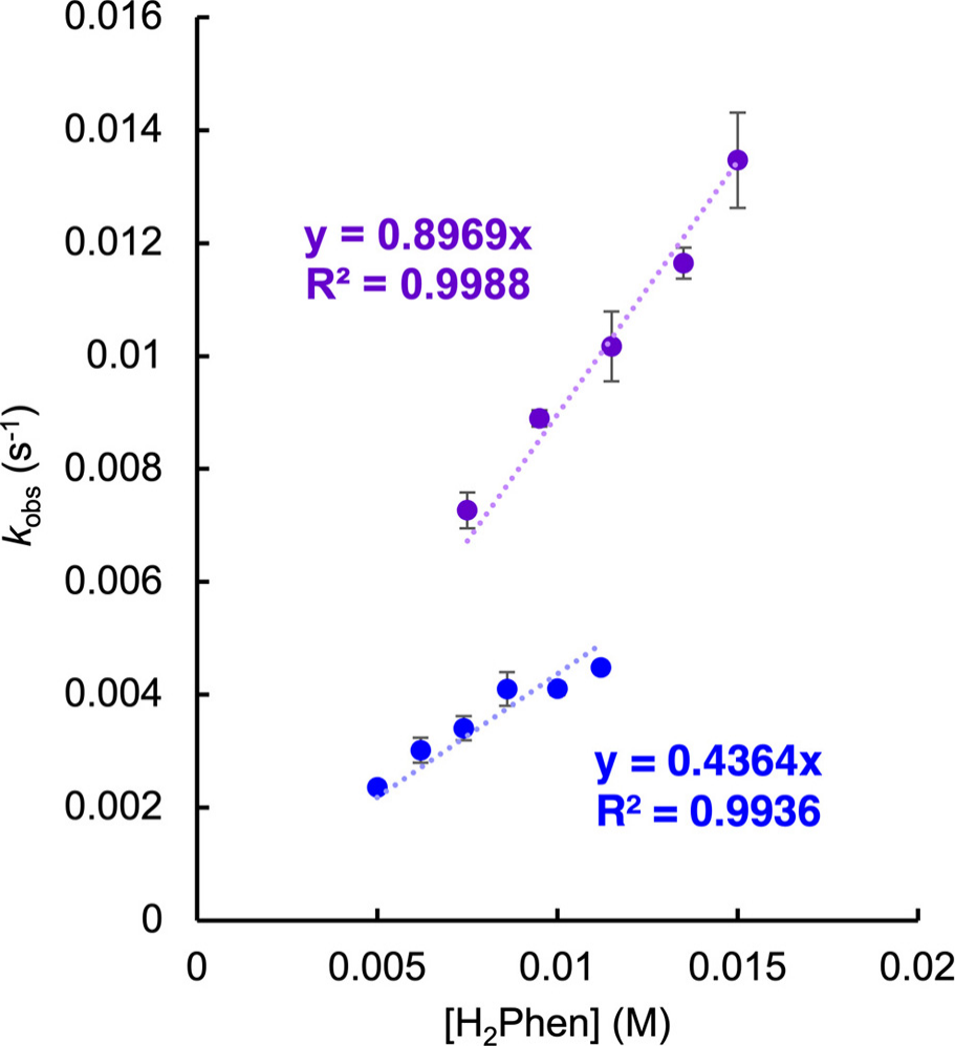
Pseudo-first order rate plots for **V**_**6**_ **O**_**6**_ **Cl** ^**–**^ (blue) and **V**_**6**_ **O**_**6**_ **SCN** ^**–**^ (purple), with excess H_2_Phen in MeCN at 25 °C. The reaction with **V**_**6**_ **O**_**6**_ **Cl** ^**–**^ was held at a constant POV-alkoxide concentration of 0.5 mM, with H_2_Phen varied between 5 and 11.2 mM. **V**_**6**_ **O**_**6**_ **SCN** ^**–**^ is held at concentration of 0.75 mM, with H_2_Phen varied between 7.5 mM and 15 mM. Linear regressions are calculated with a fixed intercept at the origin.

**Table 4:** Thermodynamic and Kinetic Parameters Describing Reactivity of **V**_**6**_ **O**_**6**_ **Cl** ^**–**^, **V**_**6**_ **O**_**6**_ **SCN** ^**–**^, and **V**_**6**_ **O**_**7**_^**–**^ with H_2_Phen

complex	**V**_**6**_ **O**_**6**_ **Cl** ^**–**^	**V**_**6**_ **O**_**6**_ **SCN** ^**–**^	**V**_**6**_ **O**_**7**_^**–**^
ox. state distribution	V^V^V^IV^_4_V^III^	V^V^V^IV^_4_V^III^	V^V^V^IV^_5_
BDFE (kcal mol^–1^)	59.4 ± 0.4	59.4 ± 0.4	59.7 ± 0.1
*k*_PCET_ (M^–1^ s^–1^@298 K)	0.0545 ± 0.005	0.112 ± 0.006	0.020 ± 0.002
Δ*H* ^⧧^ (kcal mol^–1^)	9.0 ± 0.8	7.3 ± 0.7	10.2 ± 1.3
Δ*S* ^⧧^ (cal mol^–1^ K^–1^)	–29.5 ± 2.8	–34.2 ± 2.2	–32.0 ± 4.2
Δ*G* ^⧧^ (kcal mol^–1^)	17.8 ± 1.7	17.5 ± 1.3	19.7 ± 2.5

To further understand the impact of chlorination of the Lindqvist framework, the thermochemical parameters of the transition state were evaluated. Assessment of the kinetic driving forces, enthalpy (Δ*H* ^⧧^), entropy (Δ*S* ^⧧^), and free energy of activation (Δ*G* ^⧧^) provide insights into mechanism of PCET. The enthalpy and entropy of activation provide insight into the reorganization of the solvent molecules, and overall disorder of the transition state, respectively, providing insights into the degree of H-bonding between POV-alkoxide and substrate. The temperature dependent energy of activation is then calculated at room temperature (298 K) from the enthalpy and entropy, revealing the energy barrier of the transition state.^41,42^ Lowering these values should enhance *k*_PCET_, as lower activation barriers typically lead to larger rates of reactions.^3,5,7,9,43^ Eyring analysis of the reduction of **V**_**6**_ **O**_**6**_ **Cl** ^**–**^ with H_2_Phen reveals activation parameters similar to that of the parent POV-ethoxide, **V**_**6**_ **O**_**7**_^**–**^ (Table 4, Figures S15 and S16). The similarities in the low Δ*H* ^⧧^ (**V**_**6**_ **O**_**7**_^**–**^, 10.2 ± 1.3 kcal mol^–1^; **V**_**6**_ **O**_**6**_ **Cl** ^**–**^, 9.0 ± 0.8 kcal mol^–1^) and large Δ*S* ^⧧^ (**V**_**6**_ **O**_**7**_^**–**^, −32.0 ± 4.2 cal mol^–1^ K^–1^; **V**_**6**_ **O**_**6**_ **Cl** ^**–**^, −29.5 ± 2.8 cal mol^–1^ K^–1^) suggest that H atom uptake at **V**_**6**_ **O**_**6**_ **Cl** ^**–**^ occurs through a concerted proton–electron transfer process in analogy to **V**_**6**_ **O**_**7**_^**–**^.^21,22^ Collectively the thermochemical activation parameters for **V**_**6**_ **O**_**6**_ **Cl** ^**–**^ trend toward lower values in comparison to its parent POV-ethoxide derivative. Notably, Δ*G* ^⧧^ is statistically equivalent for **V**_**6**_ **O**_**6**_ **Cl** ^**–**^ (17.8 ± 1.7 kcal mol^–1^) and **V**_**6**_ **O**_**7**_^**–**^ (19.7 ± 2.5 kcal mol^–1^), suggesting the rate enhancement is not attributed to lower kinetic barriers. Instead, this might suggest that distortion to the lattice enhances kinetics, similar to V_6_O_6_(OH_2_)­(OCH_3_)_12_.

Consistent with our qualitative assessment of rates, kinetic analyses of the reaction of H_2_Phen with **V**_**6**_ **O**_**6**_ **SCN** ^**–**^ were performed under similar conditions (see Experimental Section for details, Figure S17), revealing a *k*_PCET_ of 0.112 ± 0.006 M^–1^ s^–1^, a 2-fold enhancement from **V**_**6**_ **O**_**6**_ **Cl** ^**–**^ (*k*_PCET_ = 0.0545 ± 0.005 M^–1^ s^–1^; Figure 7, Table 4). The rate enhancement relative to **V**_**6**_ **O**_**7**_^**–**^ at **V**_**6**_ **O**_**6**_ **SCN** ^**–**^ is more pronounced than **V**_**6**_ **O**_**6**_ **Cl** ^**–**^. Unlike the TiPOV, which facilitates *k*_PCET_ enhancement from H_2_Phen under a new mechanistic regime due to the lower barrier of electron transfer, the reduction potentials of **V**_**6**_ **O**_**7**_^**–**^, **V**_**6**_ **O**_**6**_ **Cl** ^**–**^ and **V**_**6**_ **O**_**6**_ **SCN** ^**–**^ are similar, suggesting a change to electron transfer mechanism is unlikely.^25^

To confirm similar reaction mechanisms of H atom uptake at **V**_**6**_ **O**_**6**_ **Cl** ^**–**^ and **V**_**6**_ **O**_**6**_ **SCN** ^**–**^, the thermochemical parameters for the transition states were established via Eyring analysis. The values obtained for **V**_**6**_ **O**_**6**_ **SCN** ^**–**^ similarly fall within the reported valued for CPET at POV-alkoxides (Table 3; Figures S18 and S19). Surprisingly, experiments suggest that there is no major difference between the activation barriers of H atom uptake at **V**_**6**_ **O**_**6**_ **Cl** ^**–**^ (Δ*G* ^⧧^ = 17.8 ± 1.7 kcal mol^–1^) and **V**_**6**_ **O**_**6**_ **SCN** ^**–**^ (Δ*G* ^⧧^ = 17.5 ± 1.3 kcal mol^–1^). This observation suggests that the activation barrier for PCET is not the sole factor dictating the rate enhancement observed. The enthalpic and entropic contributions, instead, provide some insight. As enthalpic contribution decreases from **V**_**6**_ **O**_**6**_ **Cl** ^**–**^ (9.0 ± 0.8 kcal mol^–1^) to **V**_**6**_ **O**_**6**_ **SCN** ^**–**^ (7.3 ± 0.7 kcal mol^–1^) the rate increases (Table 4; Figure 7). Similarly, as the Δ*S* ^⧧^ values become increasingly negative from the chloride to thiocyanate doped species (**V**_**6**_ **O**_**6**_ **Cl** ^**–**^, −29.5 ± 2.8 cal mol^–1^ K^–1^; **V**_**6**_ **O**_**6**_ **SCN** ^**–**^, −34.2 ± 2.2 cal mol^–1^ K^–1^) the rate also increases (Table 3; Figure 7).

While these POV-alkoxides operate through a CPET mechanism, with a single, well-ordered bimolecular transition state, the degree of interaction between substrate and assembly can be investigated through differences in enthalpic and entropic contributions. The decrease in Δ*S* ^⧧^ from **V**_**6**_ **O**_**6**_ **Cl** ^**–**^ to **V**_**6**_ **O**_**6**_ **SCN** ^**–**^ would suggest that the thiocyanate derivative yields a more ordered transition state. This concurs with the decreased reducibility of **V**_**6**_ **O**_**5**_ **SCN** ^**–**^ compared to **V**_**6**_ **O**_**5**_ **Cl** ^**–**^, as the thiocyanate provides more electron density to the core, making a more basic surface for stronger H-bonding. Interestingly the more negative Δ*S* ^⧧^ from **V**_**6**_ **O**_**6**_ **SCN** ^**–**^ to **V**_**6**_ **O**_**6**_ **Cl** ^**–**^ is nearly perfectly thermodynamically compensated by a lower Δ*H* ^⧧^, resulting in identical Δ*G* ^⧧^ values. Previous reports suggest that smaller enthalpies of activation, coupled with larger entropies of activation reveal a stronger H-bonded pair in the transition state.^41,42^ Altogether, the kinetic parameters of this series of POV-alkoxides suggest that a more-ordered, and strongly coordinated transition state is key to accelerating the rate of reaction.

## Conclusion

The installation of an anionic dopant at the surface of a POV-alkoxide imposes a distortion to the lattice, seen in the molecular structure of **V**_**6**_ **O**_**6**_ **SCN** ^**–**^. Probing the structural impact reveals similar geometric constraints imparted by the addition of a defect site, constriction of the angle between the dopant site and equatorial plane and shortening of the axial central oxo bond distances. Investigation of the impact of these dopants on H atom uptake at the surface reveals enhanced kinetics relative to that of the fully oxygenated derivative, 2× for the Cl dopant, 5× for the SCN dopant. Analysis of the activation parameters, particularly entropy and enthalpy of activation, reveals that a more ordered transition state is responsible for accelerated reaction kinetics. Despite similar electronic and geometric structures, the impact on rate of H atom uptake is less pronounced in the case of the anionic dopants relative to the O-atom deficient POV-alkoxide. This may be due to additional steric hindrance provided by the longer alkyl chains. However, the reductive shift relative to **V**_**6**_ **O**_**6**_^**0**^ suggests the formal charge reduces the overall reducibility of the POV-alkoxide. Overall, this work provides additional evidence that structural defects (cationic, anionic dopants) yield an enhancement in rate of PCET, with the electronic impacts of the defect allowing for finer control.

## Supplementary Material



## Notes

^1^H NMR spectra, crystallographic parameters, additional CV data, BDFE­(O–H)_avg_ equilibrium data, pseudo-first order kinetic traces, and additional results for KIE determination (PDF)
