# Supplementary material for: Impact of Anionic Dopants on H Atom Uptake at Polyoxovanadate-Alkoxide Surfaces
Source: Inorg Chem. 2025 Aug 27;64(35):17935–45. doi: 10.1021/acs.inorgchem.5c02940 (PMC12421663; doi:10.1021/acs.inorgchem.5c02940)
Supplement: Supplementary file 1 [file ic5c02940_si_001.pdf]

Electronic Supporting Information

Impact of Anionic Dopants on H-atom Uptake at Polyoxovanadate-alkoxide Surfaces

M. Rebecca A. Walls, Rachel M. Meyer, William W. Brennessel, and Ellen M. Matson*

Department of Chemistry, University of Rochester, Rochester, New York 14627, USA

Corresponding Author Contact Information:

Ellen M. Matson: matson@chem.rochester.edu

Supporting Information Table of Contents

Figure S1. ESI-MS of $\mathbf{V_6O_6SCN^{1-}}$. S3

Figure S2. Full IR spectrum of $\mathbf{V_6O_6SCN^{1-}}$, neat. S3

Table S1. Crystallographic parameters for molecular structure of $\mathbf{V_6O_6SCN^{1-}}$. S4

Table S2. Bond valence sum calculations for the crystallographically independent vanadium ion in $\mathbf{V_6O_6SCN^{1-}}$ based on X-ray crystallographic data collected at 100 K. S4

Figure S3. Reaction progression of stoichiometric reduction of $\mathbf{V_6O_6Cl^{1-}}$ with H_2Phen in acetonitrile, at 25 °C. S5

Figure S4. Full FT-IR spectrum of $\mathbf{V_6O_5Cl^{1-}}$, neat. S5

Figure S5. Reaction progression of stoichiometric reduction of $\mathbf{V_6O_6SCN^{1-}}$ with H_2Phen in acetonitrile, at 25 °C. S6

Figure S6. ^{1}H NMR spectrum of $\mathbf{V_6O_5SCN^{1-}}$ in acetonitrile-d_3 at 21 °C, with region for TBA (1-4 ppm) omitted. S6

Figure S7. Full FT-IR spectrum of $\mathbf{V_6O_5SCN^{1-}}$, neat. S7

Figure S8. Cyclic voltammogram of $\mathbf{V_6O_5Cl^{1-}}$ (1 mM) in acetonitrile, with 0.1 M $TBAPF_6$ as supporting electrolyte at a scan speed of 200 mV/s. S7

Figure S9. Cyclic voltammogram of $\mathbf{V_6O_5SCN^{1-}}$ (1 mM) in acetonitrile, with 0.1 M $TBAPF_6$ as supporting electrolyte at a scan speed of 200 mV/s. S8

Figure S10. ^{1}H NMR spectra of triplicate trials establishing equilibrium between stoichiometric $\mathbf{V_6O_6Cl^{1-}}$ and H_2Azo in THF-d_8 at 21 °C. Key: blue, region for H2Azo signals (7.16 ppm, 4 H; 6.77 ppm, 6 H); yellow, Azo (7.91 ppm, 4 H; 7.58 ppm, 6 H). S8

Figure S11. ^{1}H NMR spectra of equilibrium between $\mathbf{V_6O_6Cl^{1-}}$ and 1-3 equiv. H_2Azo in THF-d_8 at 21 °C. S9

Table S3. BDFE(O-H)$_{adj}$ calculated from equilibrium reactions between $\mathbf{V_6O_6Cl^{1-}}$ and H_2Azo in THF-d_8. .... S9

Figure S12. ^{1}H NMR spectra of triplicate trials establishing equilibrium between stoichiometric **$V_6O_6SCN^{1-}$** and H_2Azo in THF-d_8 at 21 °C. S10

Figure S13. ^{1}H NMR spectra of equilibrium between **$V_6O_6SCN^{1-}$** and 1-3 equiv. H_2Azo in THF-d_8 at 21 °C. S10

Table S4. BDFE(O-H)$_{adj}$ calculated from equilibrium reactions between **$V_6O_6SCN^{1-}$** and H_2Azo in THF-d_8. ....S11

Figure S14. Psuedo-first order kinetic traces of **$V_6O_6Cl^{1-}$** (0.5 mM) and excess H_2Phen (5-11.2 mM) in acetonitrile at 25 °C. S12

Figure S15. Variable temperature (288-318 K) kinetic traces of **$V_6O_6Cl^{1-}$** (0.5 mM) and excess H_2Phen (7.4 mM) in acetonitrile. S13

Figure S16. Eyring analysis of PCET from H_2Phen to **$V_6O_6Cl^{1-}$**, plot of k_{obs} against temperature from figure S15. ....S14

Figure S17. Psuedo-first order kinetic traces of **$V_6O_6SCN^{1-}$** (0.75 mM) and excess H_2Phen (7.5-15 mM) in acetonitrile at 25 °C. S15

Figure S18. Variable temperature (288-318 K) kinetic traces of $V_6O_6SCN^{1-}$ (0.75 mM) and excess H_2Phen (11.5 mM) in acetonitrile. S16

Figure S19. Eyring analysis of PCET from H_2Phen to **$V_6O_6SCN^{1-}$**, plot of k_{obs} against temperature from figure S18. ....S17

Figure S20. Psuedo-first order kinetic traces of **$V_6O_6Cl^{1-}$** (0.5 mM) and excess D_2Phen (5-8.6 mM) in acetonitrile at 25 °C. S18

Figure S21. Kinetic isotope analysis of PCET from H_2Phen to **$V_6O_6Cl^{1-}$**, plot of k_{obs} against concentration of reductant, from figures S14 and S20........S19

Figure S22. Psuedo-first order kinetic traces of **$V_6O_6SCN^{1-}$** (0.75 mM) and excess D_2Phen (9.5-15 mM) in acetonitrile at 25 °C. S20

Figure S23. Kinetic isotope analysis of PCET from H_2Phen to **$V_6O_6SCN^{1-}$**........S21

Table S5. Summary of thermodynamic and kinetic parameters of POV-alkoxides and H_2Phen. S21

References........S22

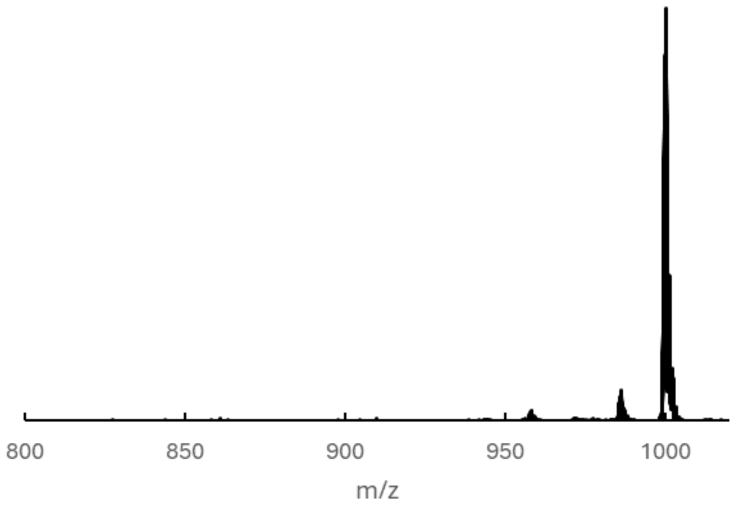

Figure S1. ESI-MS of $\mathbf{V_6O_6SCN^{1-}}$.

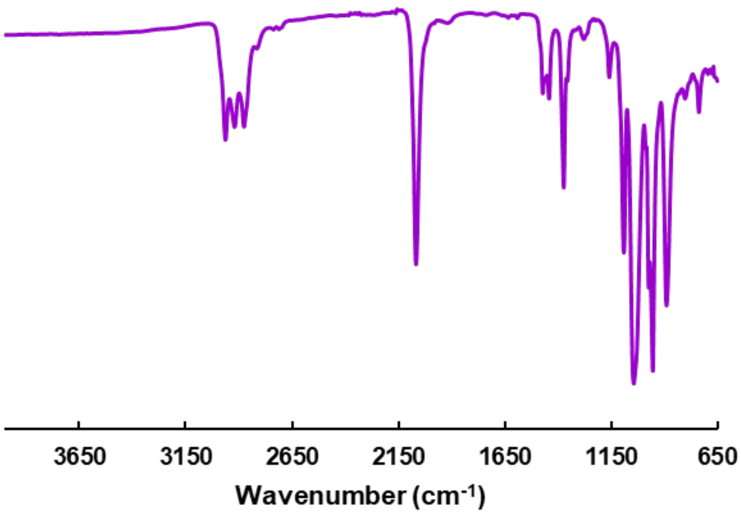

Figure S2. Full IR spectrum of $\mathbf{V_6O_6SCN^{1-}}$, neat.

Table S1. Crystallographic parameters for molecular structure of $\mathbf{V_6O_6SCN^{1-}}$.

**Compound**	$\mathbf{V_6O_6SCN^{1-}}$ CCDC 2467513
Empirical Formula	$C_{41}H_{96}N_2O_{18}SV_6$
Temperature / K	100.00(10)
Wavelength / Å	1.54184 Å
Crystal System	Monoclinic
Space Group	$P2_{1/n}$
Unit Cell Dimensions	*a* = 12.29350(10) Å; α = 90° *b* = 28.8794(2) Å; β = 93.2120(10)° *c* = 16.18960(10) Å; γ = 90°
Volume/ Å^3	5738.75(7) Å^3
Z	4
Reflections Collected	94684
Independent Reflections	12299
Completeness (theta)	99.7% (74.504°)
Goodness-of-Fit on F^2	1.016
Final *R* Indices [I>2sigma(I)]	$R1$ = 0.0375, $wR2$ = 0.0924
Largest diff. peak and hole	0.604 and -0.694 eÅ^{-3}

Table S2. Bond valence sum calculations for the crystallographically independent vanadium ion in $\mathbf{V_6O_6SCN^{1-}}$ based on X-ray crystallographic data collected at 100 K. Table reflects the results of BVS calculations using V-O bond valence parameters (r_0) for different oxidation states of vanadium.

$\mathbf{V_6O_6SCN^{1-}}$	V1	V2	V3	V4	V5	V6
V(III)	**3.101**	3.894	3.861	4.485	3.852	3.882
V(IV)	3.175	**3.987**	**3.953**	4.592	**3.944**	**3.975**
V(V)	3.412	4.250	4.217	**4.888**	4.206	4.239

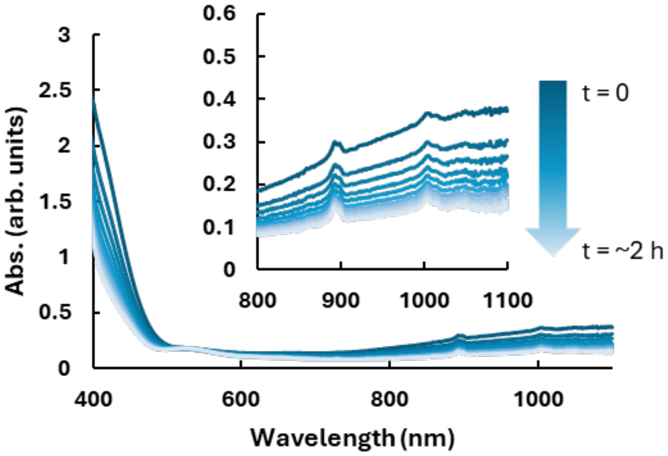

Figure S3. Reaction progression of stoichiometric reduction of $\mathbf{V_6O_6Cl^{1-}}$ with H_2Phen in acetonitrile, at 25 °C.

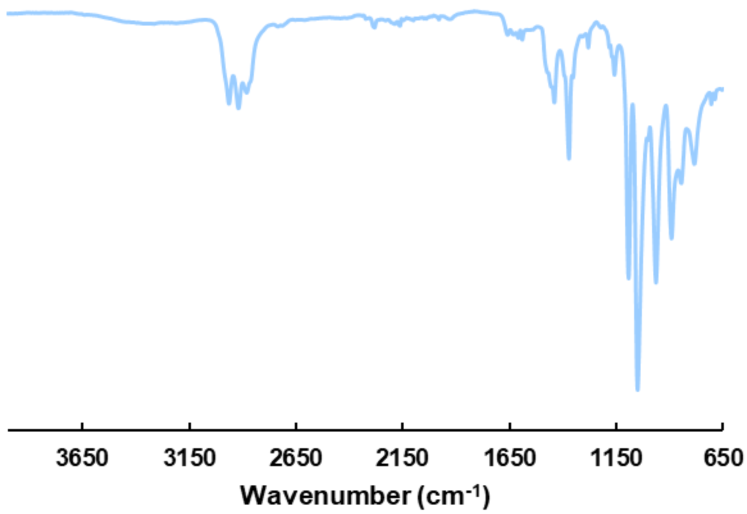

Figure S4. Full FT-IR spectrum of $\mathbf{V_6O_5Cl^{1-}}$, neat.

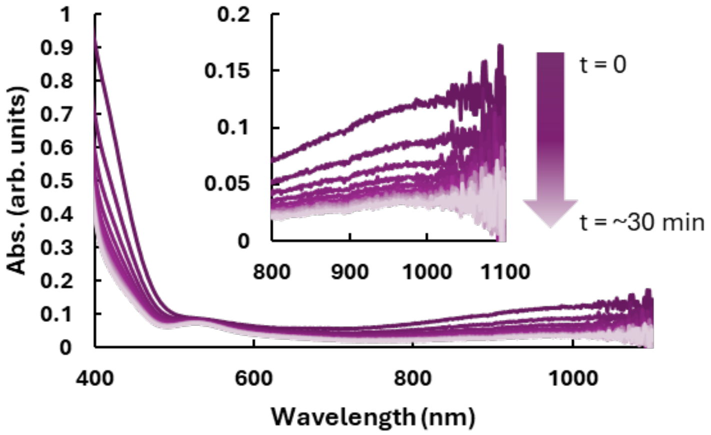

Figure S5. Reaction progression of stoichiometric reduction of $\mathbf{V_6O_6SCN^{1-}}$ with H_2Phen in acetonitrile, at 25 °C.

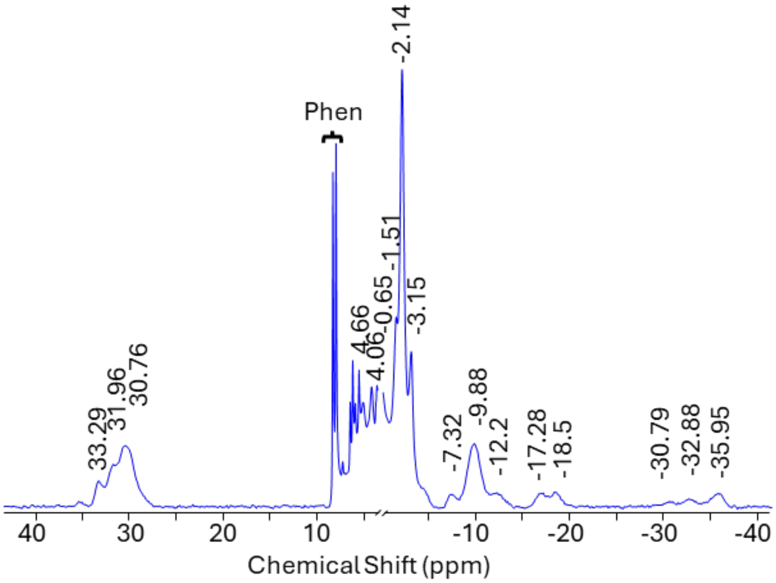

Figure S6. ^{1}H NMR spectrum of $\mathbf{V_6O_5SCN^{1-}}$ in acetonitrile-d_3 at 21 °C, with region for TBA (1-4 ppm) omitted.

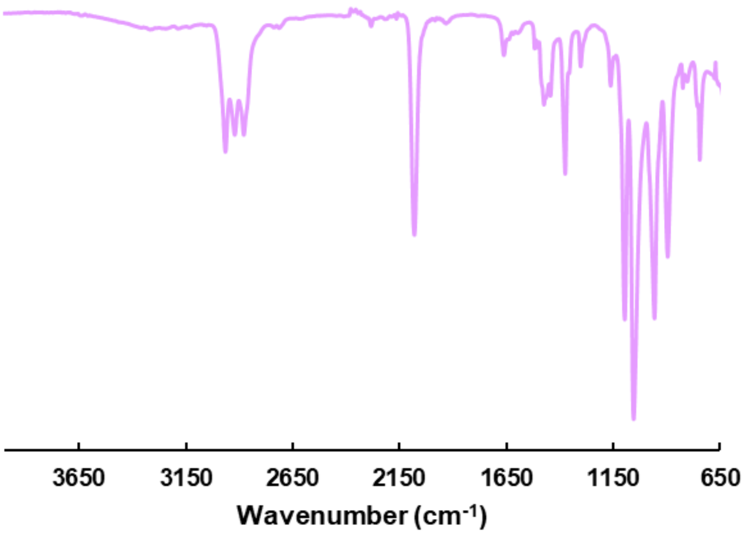

Figure S7. Full FT-IR spectrum of $\mathbf{V_6O_5SCN^{1-}}$, neat.

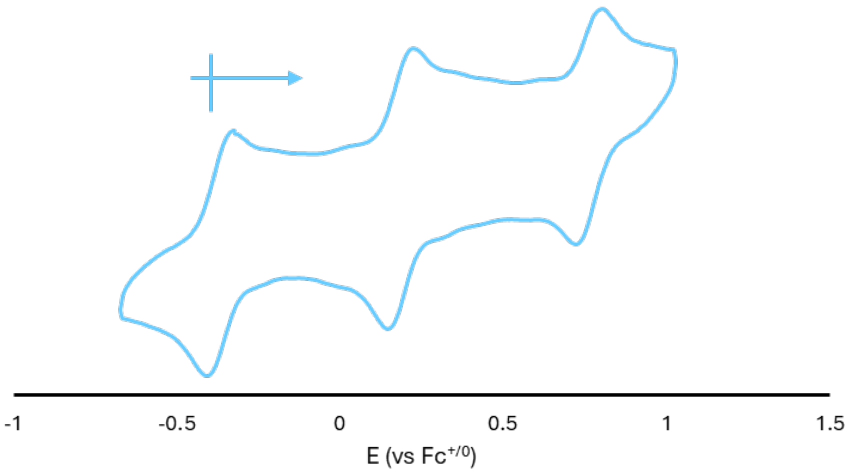

Figure S8. Cyclic voltammogram of $\mathbf{V_6O_5Cl^{1-}}$ (1 mM) in acetonitrile, with 0.1 M $TBAPF_6$ as supporting electrolyte at a scan speed of 200 mV/s.

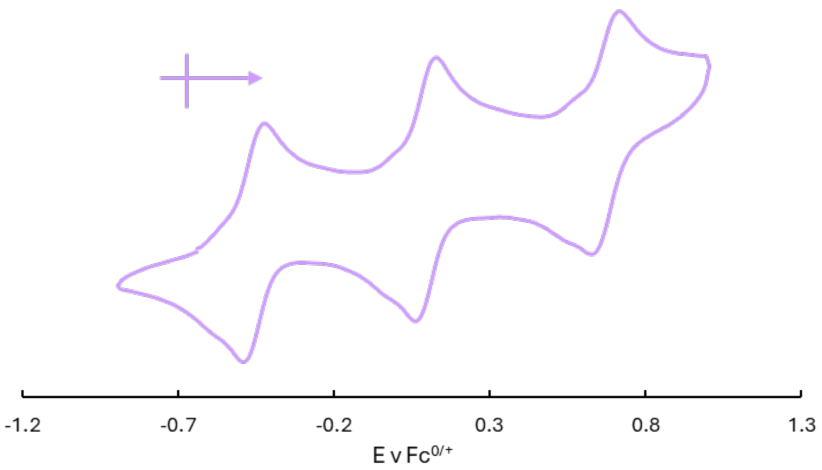

Figure S9. Cyclic voltammogram of $\mathbf{V_6O_5SCN^{1-}}$ (1 mM) in acetonitrile, with 0.1 M $TBAPF_6$ as supporting electrolyte at a scan speed of 200 mV/s.

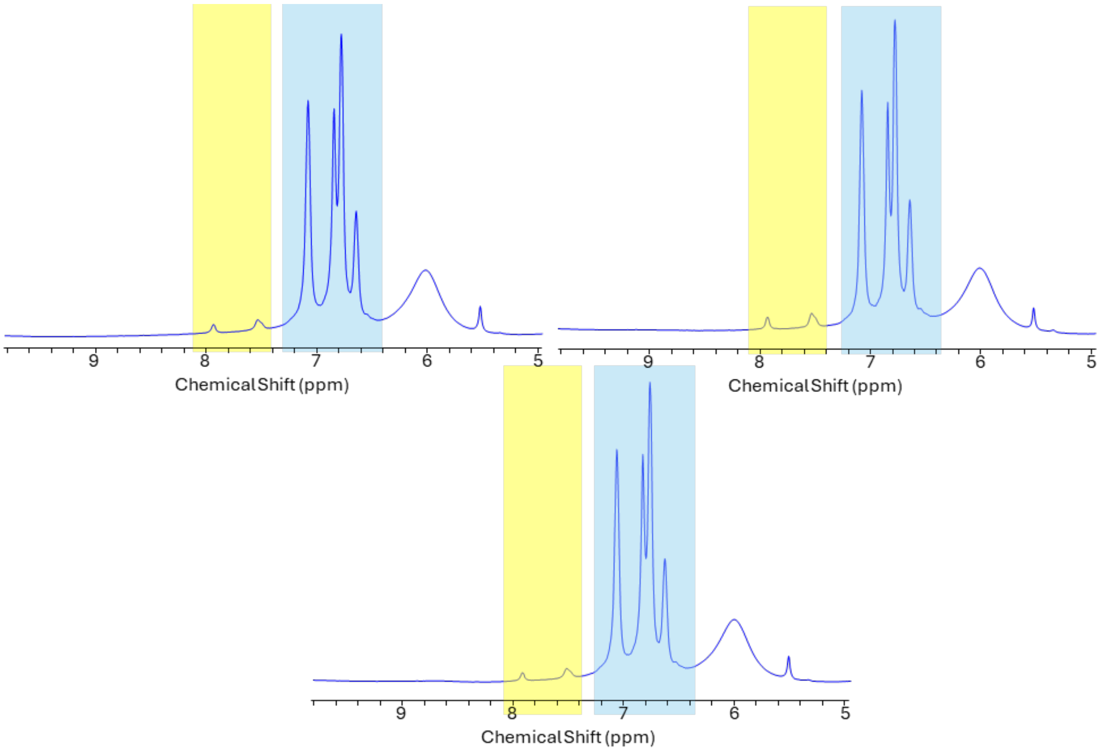

Figure S10. ^1H NMR spectra of triplicate trials establishing equilibrium between stoichiometric $\mathbf{V_6O_6Cl^{1-}}$ and H_2Azo in THF-d_8 at 21 °C. Key: blue, region for H_2Azo signals (7.16 ppm, 4 H; 6.77 ppm, 6 H); yellow, Azo (7.91 ppm, 4 H; 7.58 ppm, 6 H).

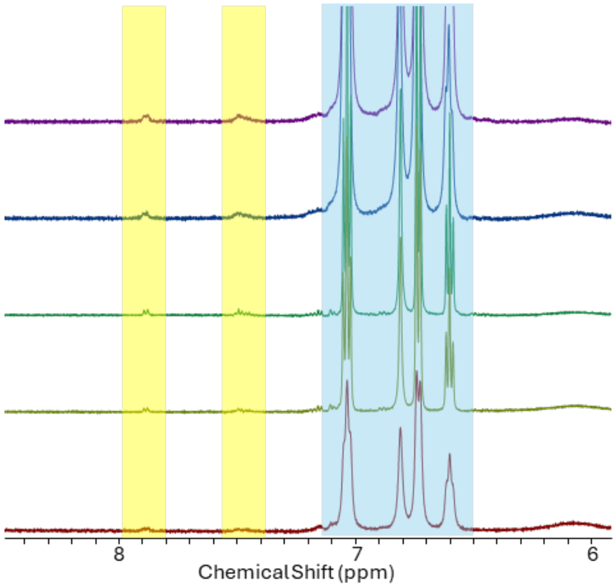

Figure S11. ¹H NMR spectra of equilibrium between $\mathbf{V_6O_6Cl^{1-}}$ and 1-3 equiv. H_2Azo in THF-d_8 at 21 °C. Key: blue, region for H_2Azo signals (7.16 ppm, 4 H; 6.77 ppm, 6 H); yellow, Azo (7.91 ppm, 4 H; 7.58 ppm, 6 H).

Table S3. BDFE(O-H)$_{adj}$ calculated from equilibrium reactions between $\mathbf{V_6O_6Cl^{1-}}$ and H_2Azo in THF-d_8.

	Hydrazobenzene (H2Azo)					Azobenzene (Azo)					
	7.16 ppm (4 H)		6.77 ppm (6 H)			7.91 ppm (4 H)		7.58 ppm (6 H)			
Trial	Integral	Relative Co	Integral	Relative Co	Avg Conc.	Integral	Relative Co	Integral	Relative Co	Avg. Conc.	BDFE
1	1	0.25	2.16	0.36	0.305	0.04	0.01	0.08	0.013333	0.011667	59.43337
2	1	0.25	2.59	0.431667	0.340833	0.04	0.01	0.09	0.015	0.0125	59.4209
3	1	0.25	2.29	0.381667	0.315833	0.04	0.01	0.11	0.018333	0.014167	59.48053
										BDFE(Avg)	59.44493
										Error	0.031455

	Hydrazobenzene (H2Azo)					Azobenzene (Azo)					
	7.16 ppm (4 H)		6.77 ppm (6 H)			7.91 ppm (4 H)		7.58 ppm (6 H)			
Equivalents	Integral	Relative Co	Integral	Relative Co	Avg Conc.	Integral	Relative Co	Integral	Relative Co	Avg. Conc.	BDFE
1	1	0.25	1.34	0.223333	0.236667	0.01	0.0025	0.01	0.001667	0.002083	58.99823
1.5	1	0.25	2.1	0.35	0.3	0.01	0.0025	0.01	0.001667	0.002083	58.928
2	1	0.25	2.06	0.343333	0.296667	0.01	0.0025	0.02	0.003333	0.002917	59.03097
2.5	1	0.25	2.05	0.341667	0.295833	0.01	0.0025	0.02	0.003333	0.002917	59.0318
3	1	0.25	2.1	0.35	0.3	0.01	0.0025	0.02	0.003333	0.002917	59.02766
										BDFE(Avg)	59.00333
										Error	0.044348

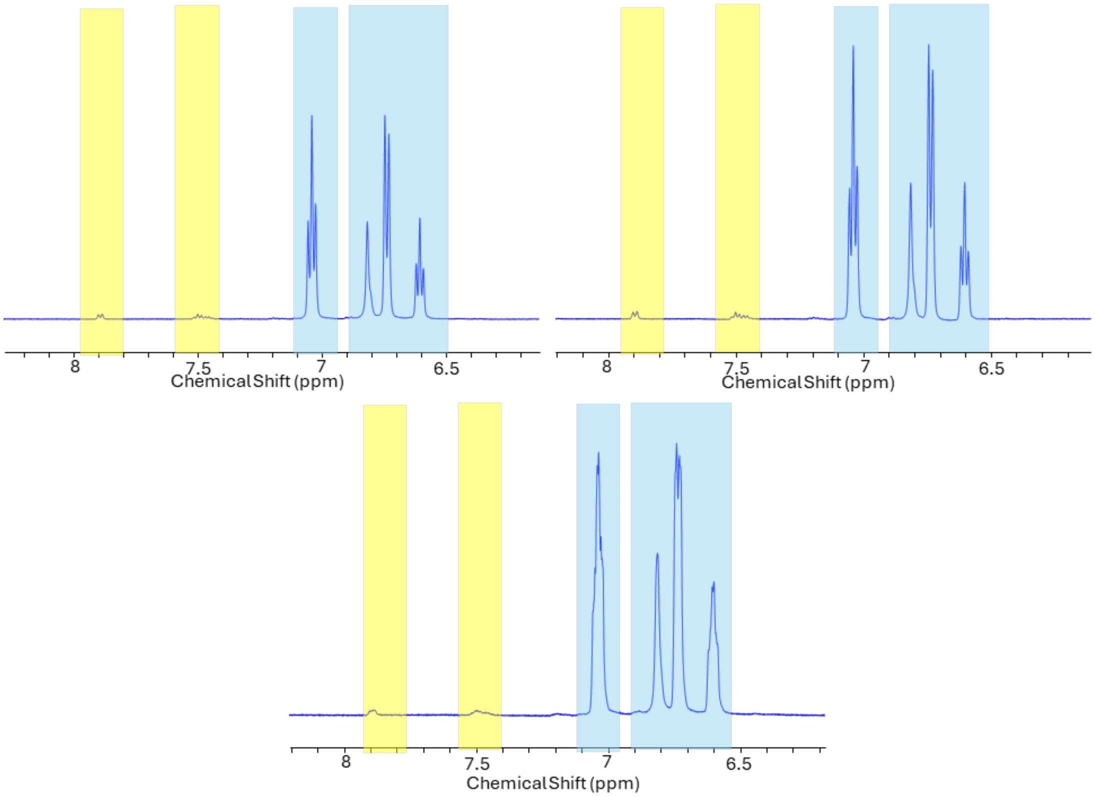

Figure S12. ^{1}H NMR spectra of triplicate trials establishing equilibrium between stoichiometric **$V_6O_6SCN^{1-}$** and H_2Azo in THF-d_8 at 21 °C. Key: blue, region for H_2Azo signals (7.16 ppm, 4 H; 6.77 ppm, 6 H); yellow, Azo (7.91 ppm, 4 H; 7.58 ppm, 6 H).

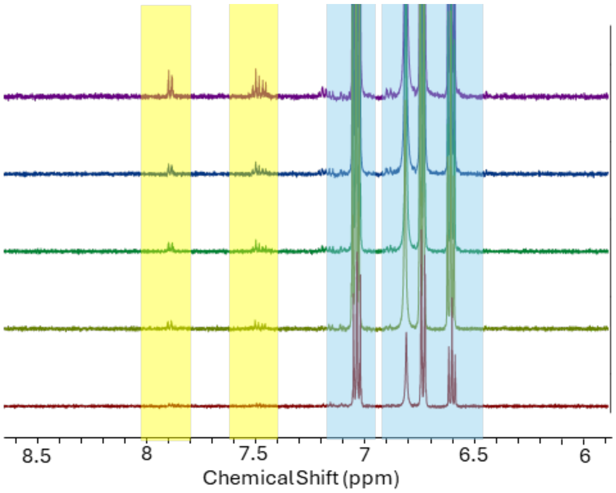

Figure S13. ^{1}H NMR spectra of equilibrium between **$V_6O_6SCN^{1-}$** and 1-3 equiv. H_2Azo in THF-d_8 at 21 °C. Key: blue, region for H_2Azo signals (7.16 ppm, 4 H; 6.77 ppm, 6 H); yellow, Azo (7.91 ppm, 4 H; 7.58 ppm, 6 H).

Table S4. BDFE(O-H)$_{adj}$ calculated from equilibrium reactions between **$V_6O_6SCN^{1-}$** and H_2Azo in THF-d_8.

METHOD A	Hydrazobenzene (H2Azo)					Azobenzene (Azo)					
	7.16 ppm (4 H)		6.77 ppm (6 H)			7.91 ppm (4 H)		7.58 ppm (6 H)			
Trial	Integral	Relative Co	Integral	Relative Co	Avg Conc.	Integral	Relative Co	Integral	Relative Co	Avg. Conc.	BDFE
1	1	0.25	1.96	0.326667	0.288333	0.21	0.0525	0.33	0.055	0.05375	59.90247
2	1	0.25	1.94	0.323333	0.286667	0.43	0.1075	0.65	0.108333	0.107917	60.11064
3	1	0.25	1.99	0.331667	0.290833	0.03	0.0075	0.05	0.008333	0.007917	59.3326
										BDFE(Avg)	59.7819
										Error	0.402786
METHOD B	Hydrazobenzene (H2Azo)					Azobenzene (Azo)					
	7.16 ppm (4 H)		6.77 ppm (6 H)			7.91 ppm (4 H)		7.58 ppm (6 H)			
Equivalents	Integral	Relative Co	Integral	Relative Co	Avg Conc.	Integral	Relative Co	Integral	Relative Co	Avg. Conc.	BDFE
1	1	0.25	1.47	0.245	0.2475	0.01	0.0025	0.02	0.003333	0.002917	59.08463
1.5	1	0.25	1.54	0.256667	0.253333	0.02	0.005	0.03	0.005	0.005	59.23738
2	1	0.25	1.55	0.258333	0.254167	0.01	0.0025	0.02	0.003333	0.002917	59.07676
2.5	1	0.25	1.57	0.261667	0.255833	0.01	0.0025	0.02	0.003333	0.002917	59.07483
3	1	0.25	1.55	0.258333	0.254167	0.01	0.0025	0.02	0.003333	0.002917	59.07676
										BDFE(Avg)	59.11007
										Error	0.071266

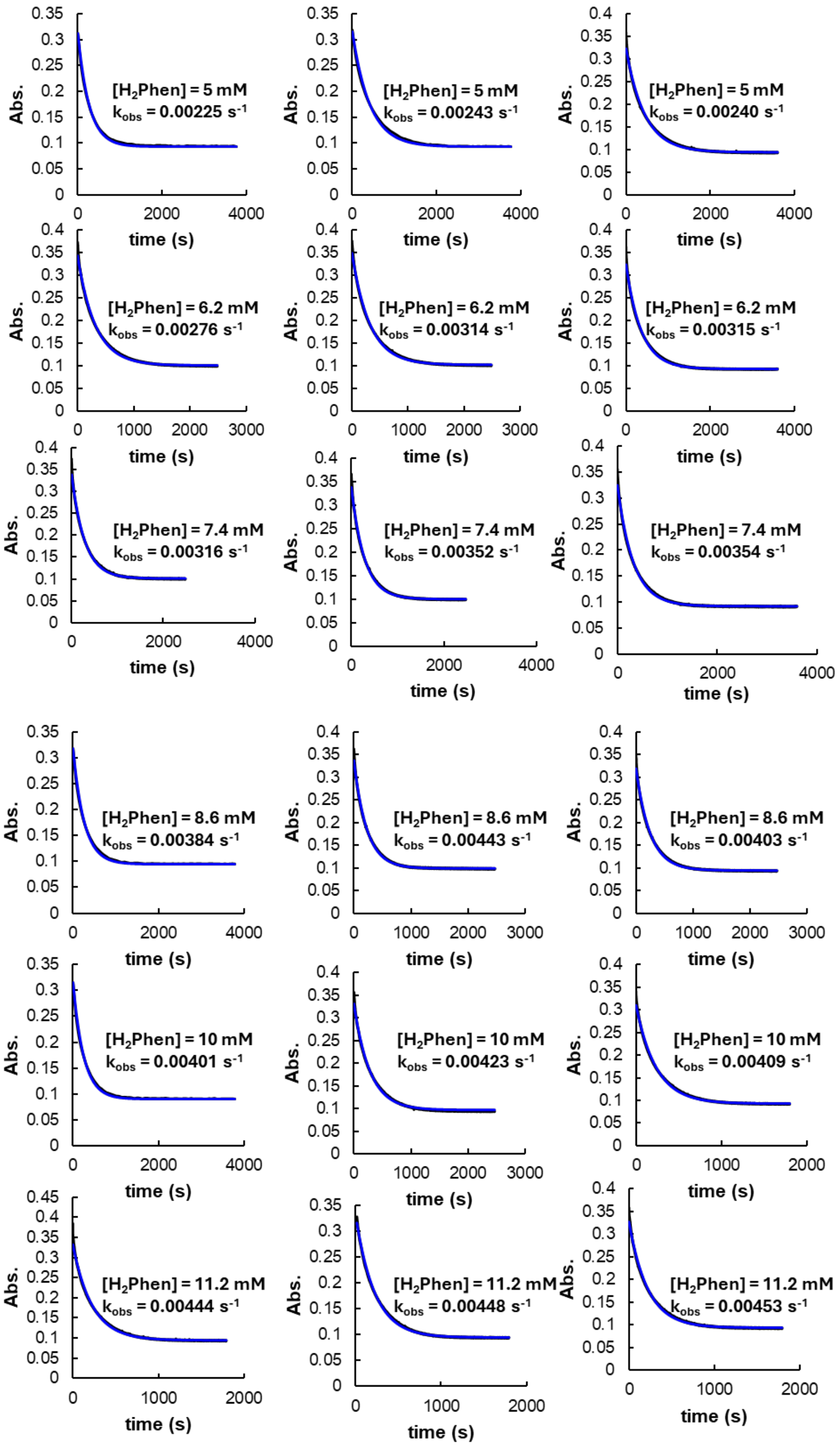

Figure S14. Psuedo-first order kinetic traces of $\mathbf{V_6O_6Cl^{1-}}$ (0.5 mM) and excess H_2Phen (5-11.2 mM) in acetonitrile at 25 °C. Concentrations and k_{obs} are listed as insets for each trial. Triplicate trials are presented.

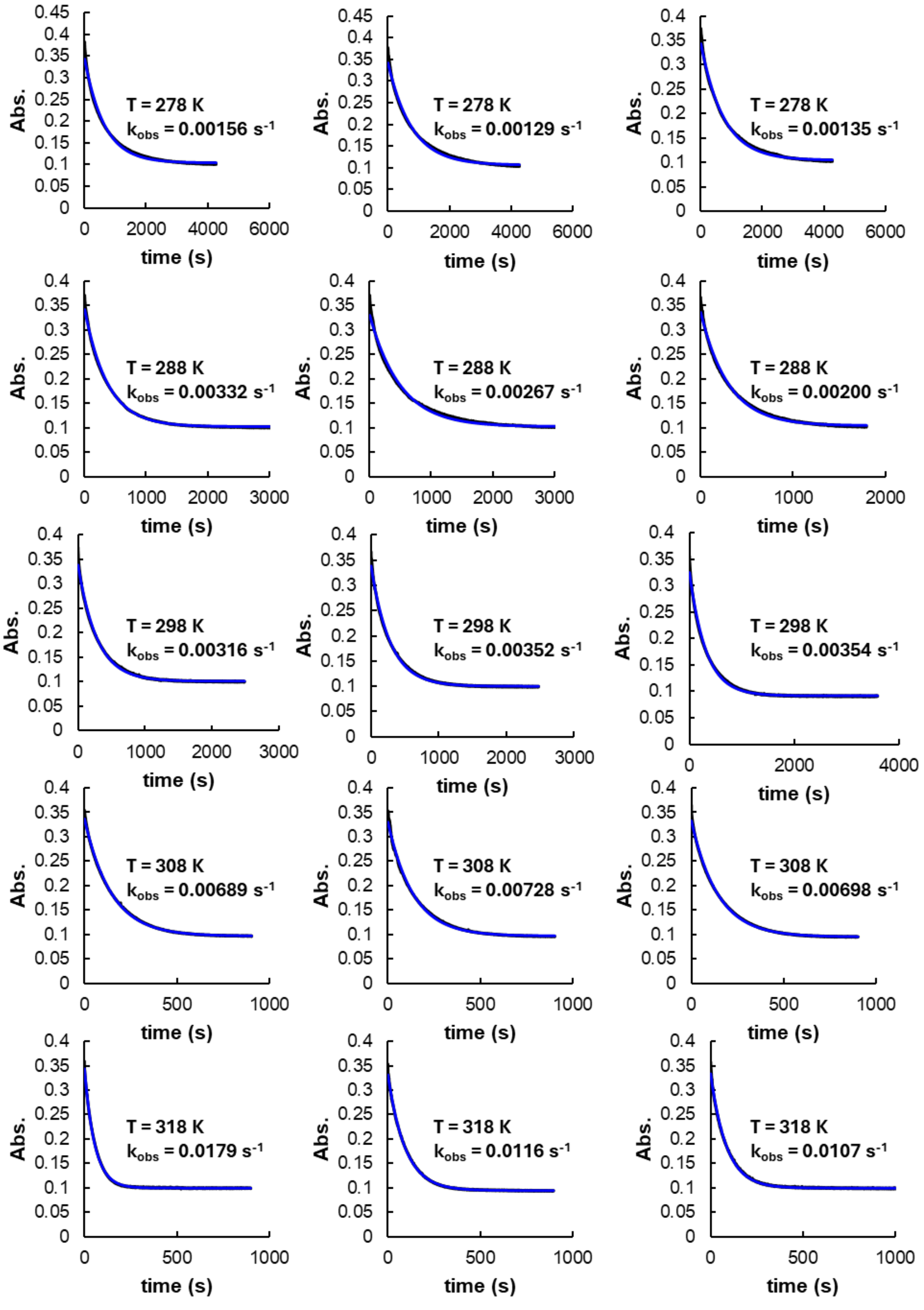

Figure S15. Variable temperature (288-318 K) kinetic traces of $\mathbf{V_6O_6Cl^{1-}}$ (0.5 mM) and excess H_2Phen (7.4 mM) in acetonitrile. Temperatures and k_{obs} are listed as insets for each trial. Triplicate trials are presented.

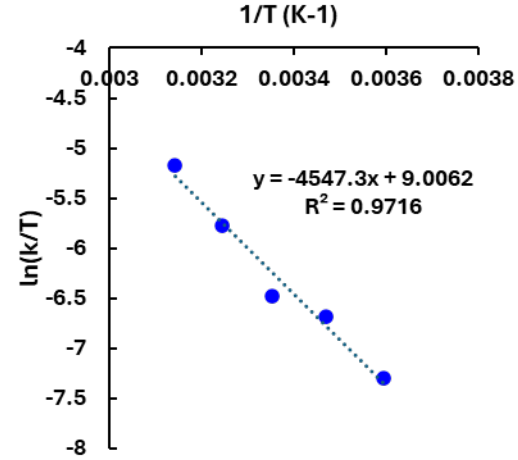

Figure S16. Eyring analysis of H-atom transfer from H_2Phen to $\mathbf{V_6O_6Cl^{1-}}$, plot of k_{obs} against temperature from **Figure S15**.

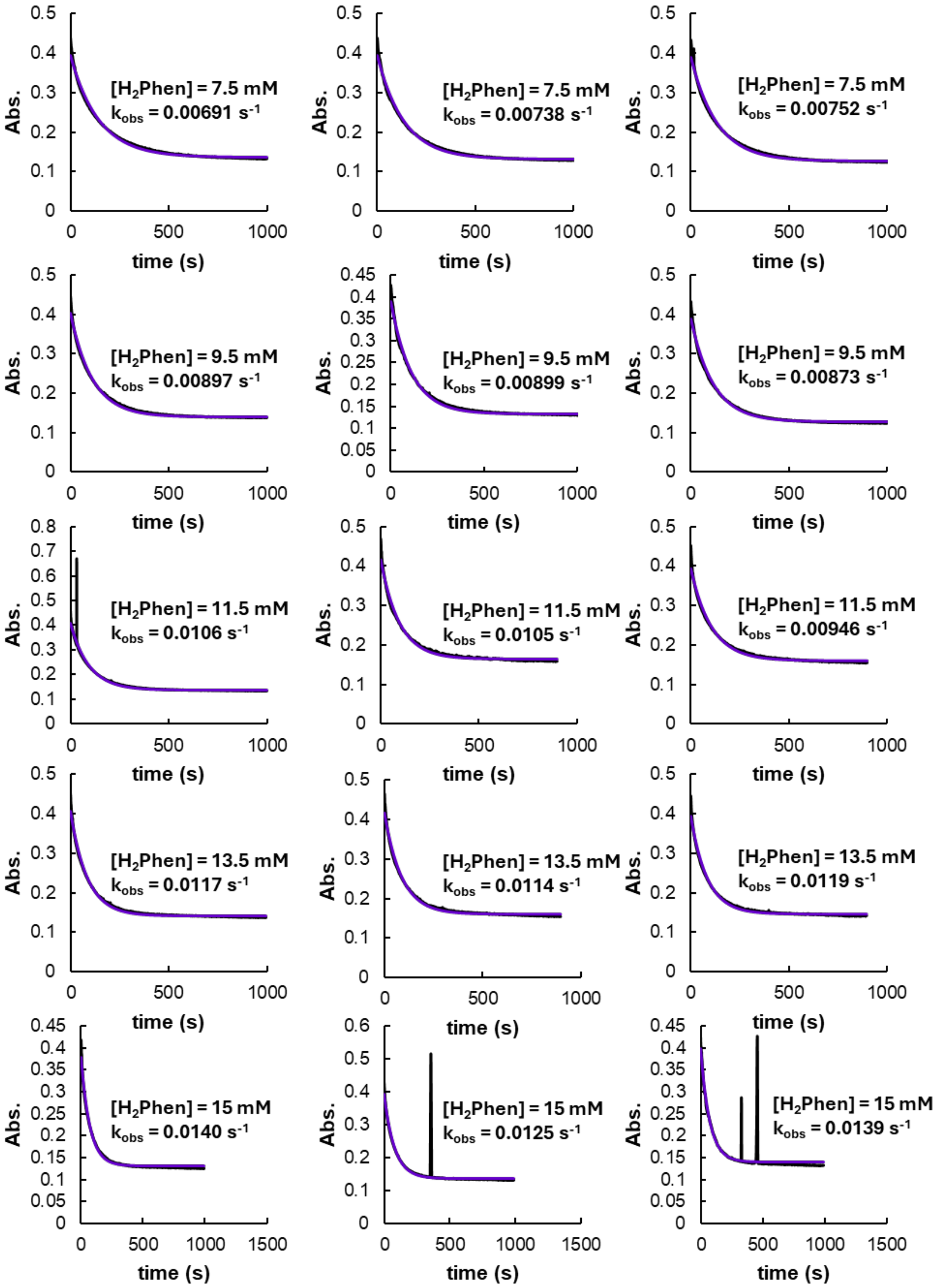

Figure S17. Psuedo-first order kinetic traces of $\mathbf{V_6O_6SCN^{1-}}$ (0.75 mM) and excess H_2Phen (7.5-15 mM) in acetonitrile at 25 °C. Concentrations and k_{obs} are listed as insets for each trial. Triplicate trials are presented.

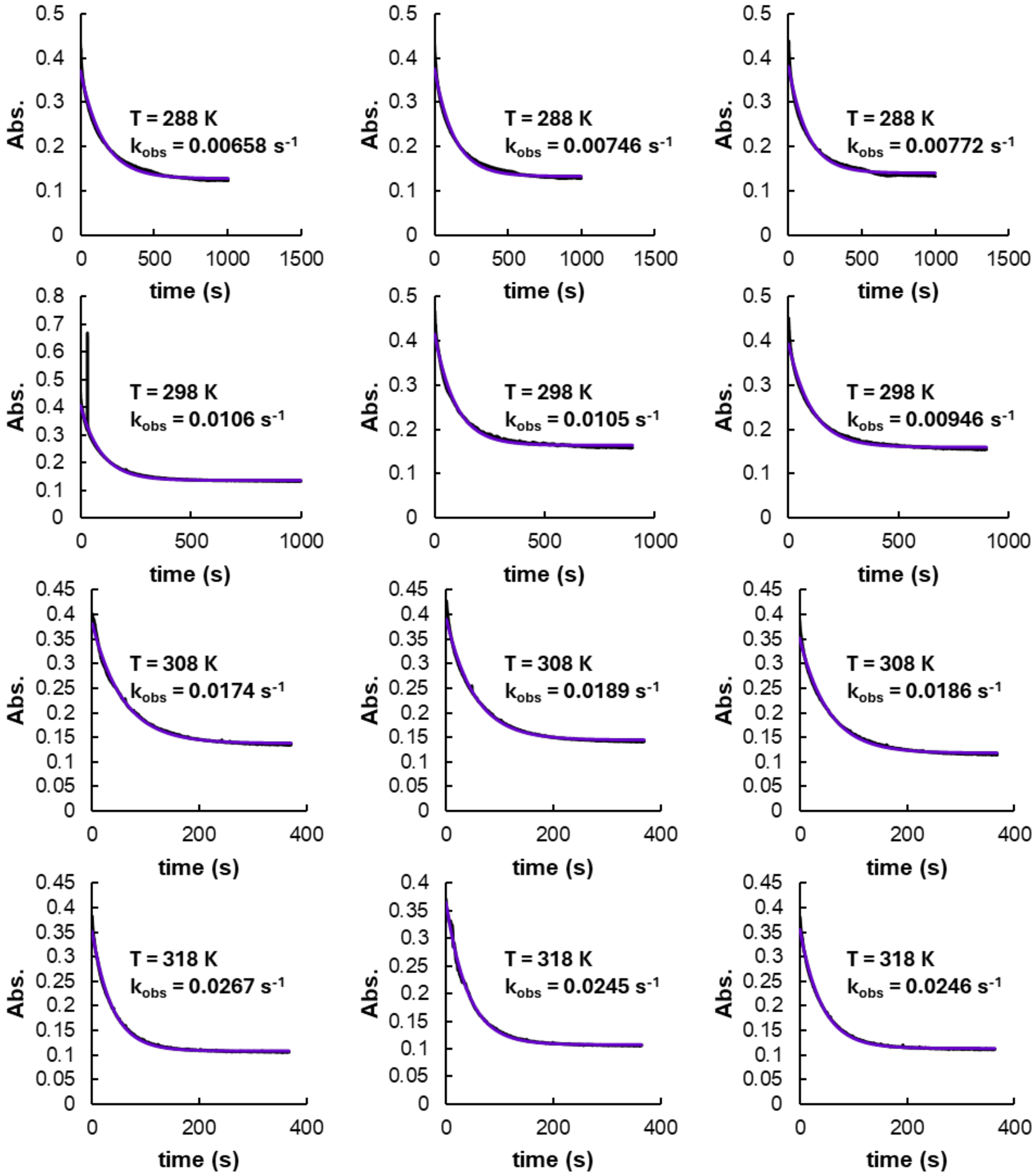

Figure S18. Variable temperature (288-318 K) kinetic traces of $\mathbf{V_6O_6SCN^{1-}}$ (0.75 mM) and excess H_2Phen (11.5 mM) in acetonitrile. Temperatures and k_{obs} are listed as insets for each trial. Triplicate trials are presented.

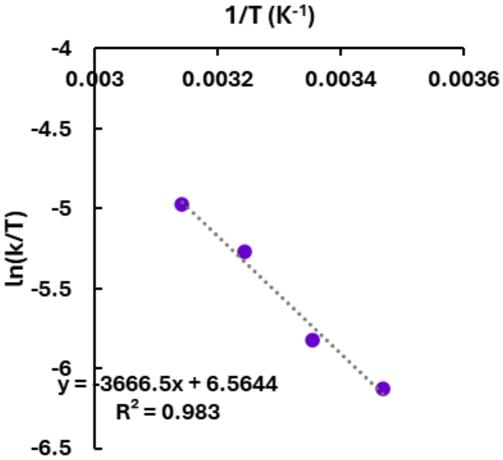

Figure S19. Eyring analysis of H-atom transfer from H_2Phen to **$V_6O_6SCN^{1-}$**, plot of k_{obs} against temperature from **Figure S18**.

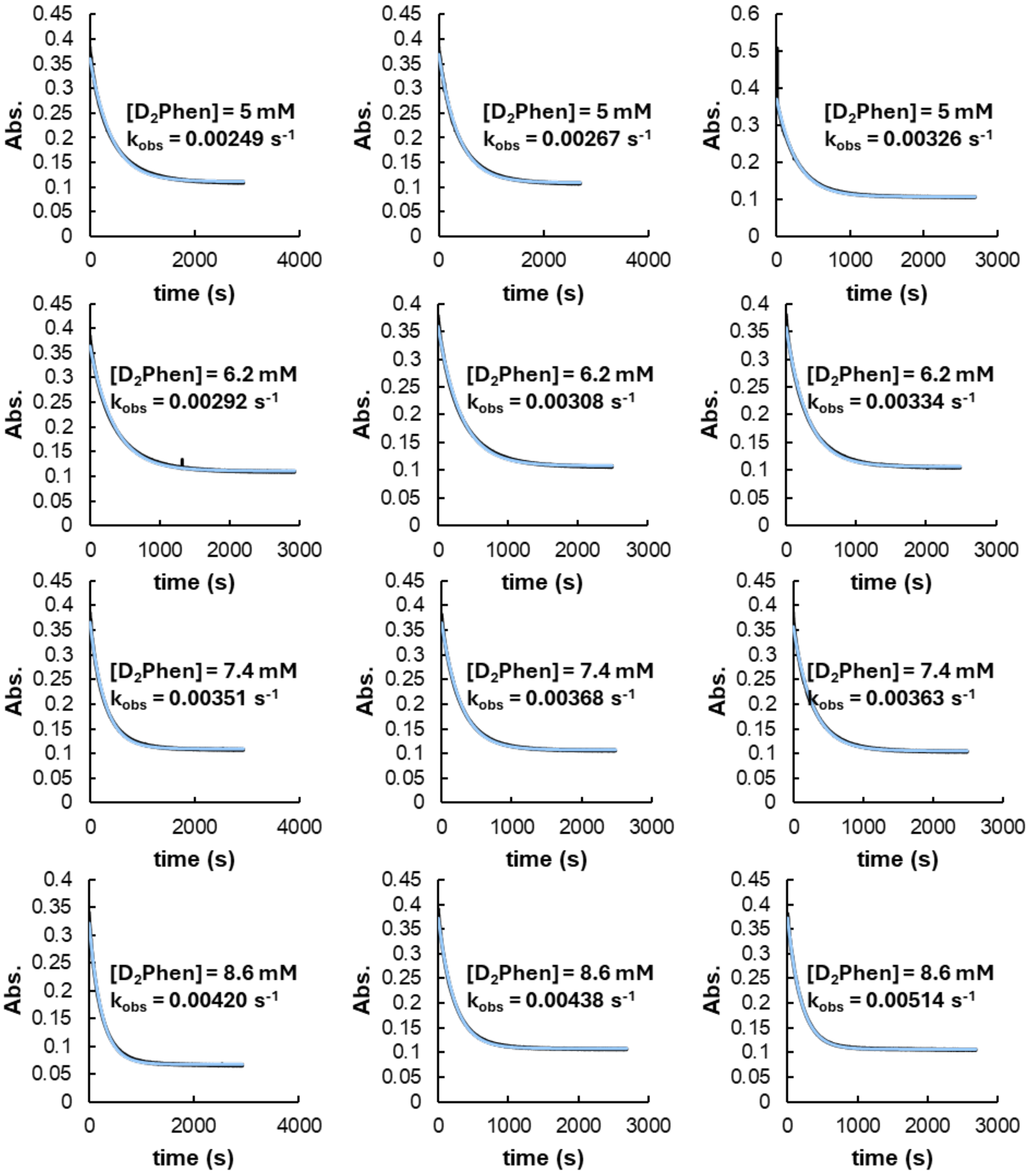

Figure S20. Psuedo-first order kinetic traces of $\mathbf{V_6O_6Cl^{1-}}$ (0.5 mM) and excess D_2Phen (5-8.6 mM) in acetonitrile at 25 °C. Concentrations and k_{obs} are listed as insets for each trial. Triplicate trials are presented.

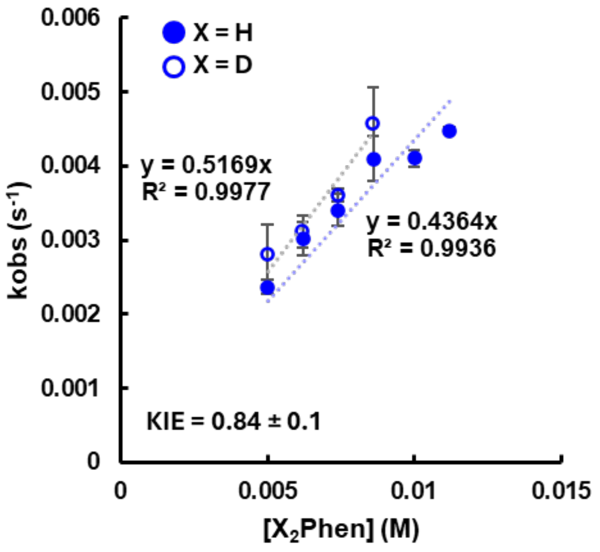

Figure S21. Kinetic isotope analysis of PCET from H_2Phen to **$V_6O_6Cl^{1-}$**, plot of k_{obs} against concentration of reductant, from **Figures S14** and **S20**.

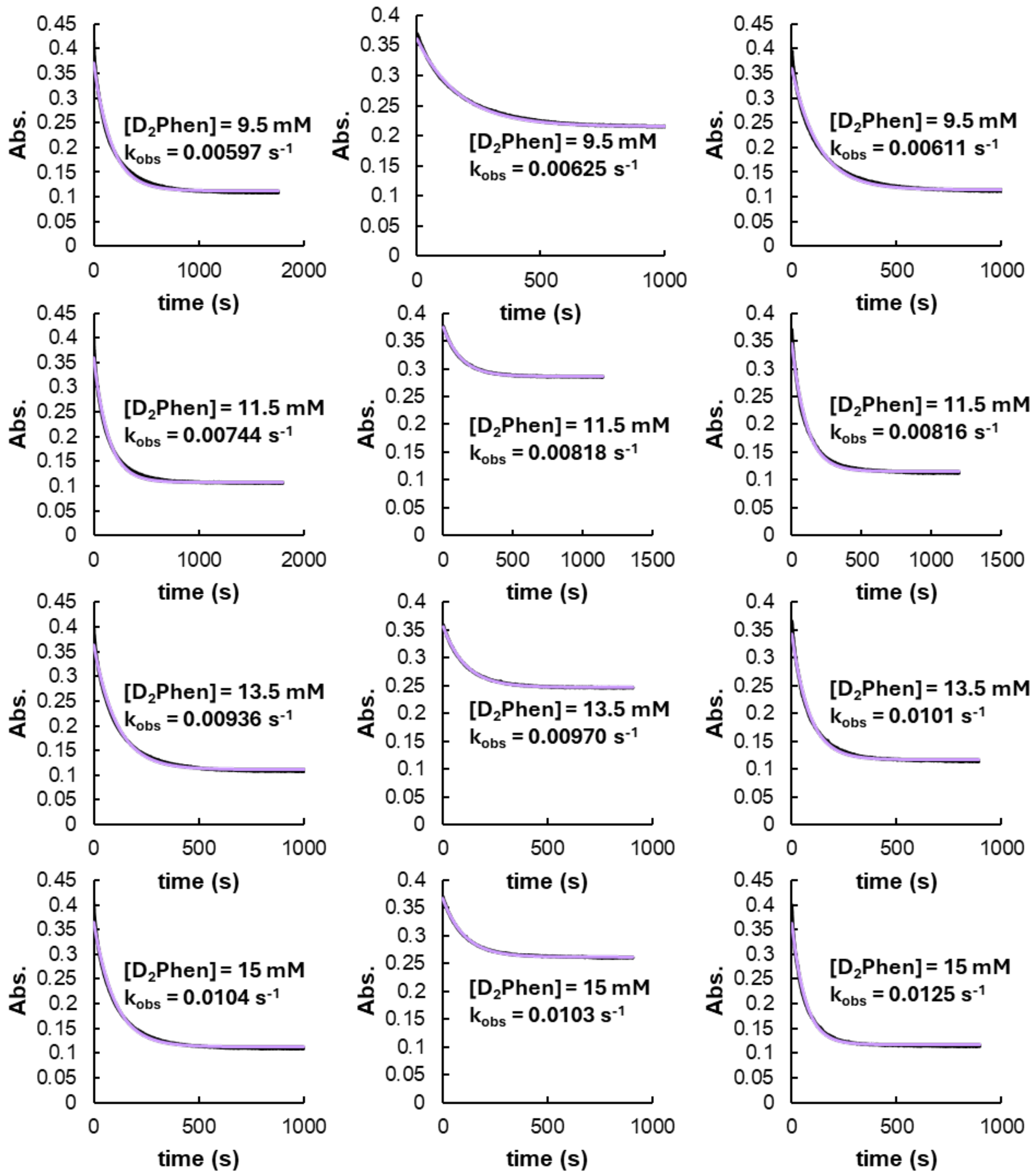

Figure S22. Psuedo-first order kinetic traces of $\mathbf{V_6O_6SCN^{1-}}$ (0.75 mM) and excess D_2Phen (9.5-15 mM) in acetonitrile at 25 °C. Concentrations and k_{obs} are listed as insets for each trial. Triplicate trials are presented.

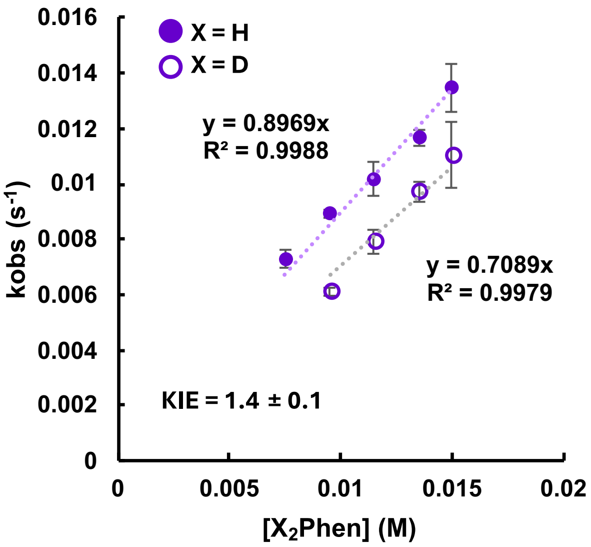

Figure S23. Kinetic isotope analysis of PCET from H_2Phen to **$V_6O_6SCN^{1-}$**, plot of k_{obs} against concentration of reductant, from **Figures S17** and **S22**.

Table S5. Summary of thermodynamic and kinetic parameters of POV-alkoxides and H_2Phen.

**Charge state**	**Cluster**	**k_{PCET}** (@298 K, $M^{-1}s^{-1}$)	**BDFE(O-H)$_{avg}$** (kcal/mol)	**ΔG‡** (@298 K, kcal/mol)	**ΔH‡** (kcal/mol)	**ΔS‡** (cal/mol·K)	**Ref.**
-2	$V_6O_8(OCH_3)_{11}$	5.6 ±	60.6 ± 0.1	16.4 ± 0.8	6.3 ± 0.4	-34.0 ± 1.5	1
-1	$V_6O_7(OCH_3)_{12}$	0.45 ±	59.9 ± 0.1	17.9 ± 1.3	6.9 ± 0.7	-37.1 ± 2.3	2
	$V_6O_7(OC_2H_5)_{12}$	0.02 ±	59.7 ± 0.1	19.7 ± 2.5	10.2 ± 1.3	-32.0 ± 4.2	
	$V_6O_7(OC_3H_7)_{12}$	0.029 ±	59.7 ± 0.1	19.5 ± 2.8	9.5 ± 1.4	-33.7 ± 4.7	
	$V_6O_7(OC_4H_9)_{12}$	0.022 ±	59.7 ± 0.1	19.8 ± 2.7	9.8 ± 1.3	-33.6 ± 4.5	
0	$V_6O_7(OCH_3)_{12}$	0.14 ± 0.05	62.3 ± 0.1	18.7 ± 1.7	7.8 ± 0.8	-31 ± 3.3	3
	$V_6O_6(OCH_3)_{12}$	19.6 ± 3.2	60.7 ± 0.1	14.4 ± 2.6	4.3 ± 1.2	-33.9 ± 4.8	

References

(1) Schreiber, E.; Brennessel, W. W.; Matson, E. M. Regioselectivity of concerted proton–electron transfer at the surface of a polyoxovanadate cluster. *Chemical Science* **2023**, *14*, 1386-1396. DOI: 10.1039/D2SC05928B.

(2) Peter, C. Y. M.; Schreiber, E.; Proe, K. R.; Matson, E. M. Surface ligand length influences kinetics of H-atom uptake in polyoxovanadate-alkoxide clusters. *Dalton Transactions* **2023**, *52*, 15775-15785. DOI: 10.1039/D3DT02074F.

(3) Cooney, S. E.; Schreiber, E.; Brennessel, W. W.; Matson, E. M. Accelerated rates of proton coupled electron transfer to oxygen deficient polyoxovanadate–alkoxide clusters. *Inorganic Chemistry Frontiers* **2023**, *10*, 2754-2765. DOI: 10.1039/D3QI00129F.
